# Lung cancer cells upregulate stearoyl-CoA desaturase 1 in microglia by activating the STAT3 pathway to change microglial inflammatory response in lung-to-brain metastases

**DOI:** 10.1038/s41419-025-08003-2

**Published:** 2025-10-06

**Authors:** Peng Chen, Yufeng Zuo, Minghuan Wang, Rui Liu, Ying Li, Ziyue Wang, Hao Huang, Xiang Luo, Wei Wang, Yingying Wu, Zhiyuan Yu

**Affiliations:** 1 Department of Neurology, Tongji Hospital, Tongji Medical College, Huazhong University of Science and Technology, Wuhan, Hubei China; 2 Department of Anatomy, School of Basic Medicine, Anhui Medical University, Hefei, Anhui China; 3 Hubei Key Laboratory of Neural Injury and Functional Reconstruction, Huazhong University of Science and Technology, Huazhong, China; 4 Department of Oncology, Tongji Hospital, Tongji Medical College, Huazhong University of Science and Technology, Wuhan, Hubei China

**Keywords:** Microglia, Cancer metabolism, CNS cancer, Cancer microenvironment

## Abstract

Lung cancer brain metastases have been considered a terminal disease stage with limited treatment options. Many studies have shown that microglia as the resident macrophages in the brain form a major component of the brain immune system, and the lipid metabolism of macrophages in the tumor microenvironment could directly influence tumor progression. However, limited studies have explored the regulatory role of lipid metabolism on microglia in brain metastases. In this study, we found that lung cancer cells could promote microglia to express stearoyl-CoA desaturase 1 (SCD1) and accumulate lipid droplets. Increased activity of SCD1 in microglia reduced its response to inflammatory stimuli and promoted the proliferation of cancer cells. Notably, the treatment of tumor-bearing mice with an SCD1 inhibitor combined with an inhibitor of colony-stimulating factor 1 receptor (CSF1R) significantly reduced brain metastases. Mechanistically, we demonstrated that lung cancer cells activated the STAT3 signaling pathway in microglia leading to increased SCD1 expression. In conclusion, our findings indicate that lung cancer cells activate the microglial STAT3-SCD1-lipid metabolism-inflammatory response pathway in the brain tumor microenvironment and present a potential new strategy for treating brain metastases of lung cancer.

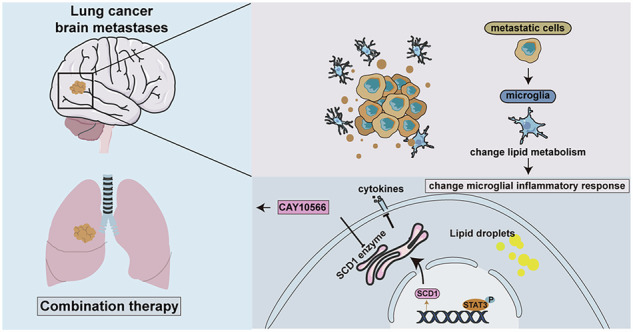

## Introduction

Brain metastases (BrM) are the most common type of intracerebral tumor in adults [1]. Although innovations in tumor research have continuously improved the quality of life and survival of such patients, brain metastases still account for about 20% of brain tumor deaths [2]. The most common primary tumor that metastasizes to the brain is lung cancer, which accounts for 39 to 56% of all brain metastases [3–5]. Indeed, up to half of lung cancer patients will develop brain metastases [6, 7], among whom non-small cell lung cancer (NSCLC) is the most common subtype [8–10]. Patients with brain metastases may manifest various symptoms, leading to a sharp decline in quality of life and hindering their eligibility for further treatment [11–14]. Despite notable progress in understanding the mechanisms of brain metastases, the prognosis of lung cancer patients with brain metastases remains poor, and effective treatment options for brain metastases are still limited [15]. Therefore, it is crucial to gain a deeper understanding of the mechanisms of lung cancer brain metastases and to identify effective therapeutic targets for brain metastases.

In established brain metastases, microglia and macrophages, collectively referred to as tumor-associated macrophages (TAMs), constitute the most abundant non-tumor cell component, accounting for 30% of the entire tumor mass [16, 17]. Microglia, as resident immune cells in the central nervous system (CNS), normally detect and eliminate any damage that may disrupt normal brain function [18, 19]. However, when recruited to brain metastatic sites, microglia may undergo a phenotypic switch to either tumor-supportive or non-supportive cells, depending on the microenvironmental context and disease conditions [20]. Previous studies have reported dense clustering of activated microglia surrounding brain metastatic lesions [21, 22]. Microglia can rapidly respond to metastatic cancer cells in the brain and influence various processes such as tumor cell colonization, migration, and proliferation, as well as regulate the immune-suppressive microenvironment, thereby playing a crucial role in the formation of brain metastases [21]. In the tumor microenvironment, lipid metabolism plays a unique and complex role in metastasis, and immune regulation [23, 24]. Studies have revealed that lipids in the tumor microenvironment exhibit remarkable diversity in terms of their types and functions, exerting opposing effects in promoting or impeding tumor growth [25]. Notably, during the differentiation process of TAMs, their lipid metabolism undergoes reprogramming, which results in the formation of aberrant metabolites that directly influence TAM polarization [26]. As a pivotal enzyme in the process of fatty acid metabolism, stearoyl-CoA desaturase 1 (SCD1) catalyzes the rate-limiting step in the synthesis of monounsaturated fatty acids (MUFAs), including palmitoleic acid (16:1 n-7) and oleic acid (18:1 n-9) from saturated fatty acids such as palmitic acid (16:0) and stearic acid (18:0) [27]. Fatty acids serve as vital constituents of cell membranes, and the balance between unsaturated and saturated fatty acids can profoundly impact membrane fluidity and signal transduction, thereby participating in the regulation of cellular growth and differentiation [28]. Extensive research has demonstrated that SCD1 exerts a significant influence on various cellular functions involved in carcinogenic signaling pathways [29, 30]. In microglia, alterations in SCD1 expression have been shown to affect the inflammatory state and phagocytic activity of microglia [31, 32]. Moreover, upregulation of SCD1 expression can lead to intracellular lipid droplet accumulation [33, 34]. Some studies have found that the accumulation of lipid droplets in TAMs promotes immunosuppressive phenotypes [35, 36]. However, limited studies have explored the regulatory role of SCD1 within the tumor immune microenvironment on the function of TAMs and their anti-tumor immune responses.

In this study, we investigated the upregulation of SCD1 expression with the accumulation of lipid droplets in microglia within the microenvironment of lung cancer brain metastases. Increased expression of SCD1 in microglia attenuated their responsiveness to inflammatory stimuli and facilitated the proliferation of lung cancer cells. Conversely, suppression of SCD1 enzymatic activity impeded the impact of cancer cells on the inflammatory responsiveness of microglia and altered their pro-tumor proliferative effect. Notably, we found that combination therapy comprising an SCD1 inhibitor and a CSF1R inhibitor significantly reduced tumor burden in mouse brain metastasis models. Additionally, we discovered that lung cancer cells robustly activated the STAT3 signaling pathway in microglia, resulting in enhanced expression of SCD1 protein. We thus established a STAT3-SCD1-lipid metabolism-inflammatory response pathway occurring in microglia residing in the brain metastasis microenvironment and presents a potential new strategy for treating brain metastases of lung cancer.

## Materials and Methods

### Human samples

Paraffin-embedded brain tumor sections from fifteen patients with lung cancer brain metastasis were obtained from the Department of Pathology at Tongji Hospital, Tongji Medical College, Huazhong University of Science and Technology. These specimens had been obtained by surgical resection and were diagnosed by pathologists as brain metastasis of non-small cell lung cancer, and associated basic clinical data and pathological slides are accessible. Written informed consent was obtained from all participating patients and/or their next of kin. This study was approved by the human research ethics committee at Tongji Hospital, Tongji Medical College, Huazhong University of Science and Technology (TJ-IRB20210222).

### Cells and in vitro culture

Human microglial cells HMC3 (American Type Culture Collection (ATCC), USA) were cultured in MEM (Gibco, USA) with 10% fetal bovine serum (FBS, Every Green, China). The human lung cancer A549 cells and NCI-H446 cells were purchased from the China Center for Type Culture Collection (CCTCC, China) and maintained in RPMI-1640 (Gibco, USA) with 10% FBS and 100 U/mL penicillin-streptomycin (Biosharp, China). A549-BrM was generously gifted by Dr. Yuan and cultured as previously described [22]. Cells were grown at 37 °C in a humidified incubator containing 5% CO_2_.

### Co-Culture Experiment and Conditioned Media Preparation

For microglia-tumor cell co-culture studies, microglia were seeded in 6-well plates (for protein/RNA analysis) or 24-well plates (for immunofluorescence) and allowed to adhere until reaching 50–70% confluence. Cells were then serum-reduced in a 2% FBS-containing medium for 8 hours to synchronize metabolic states. Concurrently, tumor cells were resuspended in a 2% FBS medium and seeded onto 0.4 µm pore polyester membrane transwell inserts (Corning, USA). These inserts were placed above the pre-treated microglia cultures. After 24 hours of co-culture, the microglia in the lower chamber were harvested for downstream analyses.

For the collection of conditioned media from lung cancer cells, the cells were cultured to 50% confluence and then incubated in the fresh medium for 48 hours. The supernatants were sequentially centrifuged at 300 g for 10 min at 4 °C, followed by a second centrifugation at 2000 g for 10 min at 4 °C to remove cells and debris. The clarified media were sterilized using 0.45 μm filters (Millipore, USA) and stored in aliquots at −80 °C.

### Mice models

The brain metastases model was established as described previously [37]. In brief, male Balb/c nude mice (GemPharmatech, China) aged 6-8 weeks were randomly divided into groups and injected with 2 × 10^5^ luciferase-labeled A549-BrM cells into the left cardiac ventricle in 100 µL PBS. To observe the subsequent formation of tumors in the brain, mice were intraperitoneally injected with D-luciferin (150 mg/kg, Gold Biotechnology, USA) and then imaged and analyzed using the Xenogen IVIS system. For in vivo treatment, mice were randomized to receive either CAY10566 (3 mg/kg, Cayman Chemicals, USA) dissolved in DMSO and diluted in corn oil or vehicle control (DMSO diluted in corn oil without the drug) via oral gavage daily for three weeks. For CSF1R treatment, mice were administered dietary PLX5622 (1200 mg/kg feed, Biopike, China) starting 1 week before the injection of tumor cells and continuing until the end of the experiment. During the process of analyzing the image result data of brain metastases in mice, the evaluators were unaware of the allocation situation of each group. All animal experiments were performed following the Ethics Committee of the Institutional Animal Care and Use Committee of Tongji Medical College, Huazhong University of Science and Technology (IACUC NO.3212).

### Western blotting

The cells were lysed in RIPA buffer with a proteinase inhibitor (Beyotime Biotechnology, China) on ice, and the protein concentration was detected using the BCA assay (Servicebio, China). Aliquots of microglia proteins were loaded and separated using SDS-PAGE 10% Tris-HCl gels and transferred to NC membranes (Merck Millipore, USA). The membranes were blocked with 5% BSA (Sigma-Aldrich, USA) for 1 h at room temperature and then incubated overnight with primary antibodies, including SCD1, STAT3 (1:1000, Abcam, USA) and β-actin (1:1000, Abclonal, China), followed by incubated with HRP-conjugated secondary antibodies for 1 h at room temperature (1:5000, Jackson ImmunoResearch, USA). The blots were developed with a western blotting detection reagent (Advansta, USA), and a protein marker (ThermoFisher Scientific, USA) was used to observe the blots’ positions. The obtained images were quantitatively analyzed with Image J software.

### Fatty acid analysis

To detect fatty acid content in cultured cells, we collected cells and rapidly froze them in liquid nitrogen. After drying and weighing the cell samples, 2 mL 5% concentrated methanol sulfate solution and 25 μL 0.2% BHT-CH3OH were added to the tubes. The samples were bathed in a water bath at 90 °C for 90 min. After removal from the water bath, we added 2 mL saturated salt solution and 1 mL n-hexane to each sample and centrifuged at 3,500 rpm for 5 min. At the same time, the reference standards and samples were added to the sample bottle. 38 fatty acids were used as the internal standards. Peak retention times were identified by injecting known standards. All data were analyzed by FID Chem Station and ACD/Spectrus 2015 software.

### Immunofluorescent staining

For immunofluorescent staining, we added PBS solution containing 0.3% Triton X-100 to disrupt the cell membranes for 15 min. The cells were then incubated with 400 μL of immunofluorescence-blocking solution (Beyotime Biotechnology, China) at room temperature for one hour. After that, they were covered with the IBA1 (1:400, WAKO, Japan) or TMEM119 (1:200, Sigma-Aldrich, USA) to visualize microglia, HuNu (1:500, Merck Millipore, USA) to label human-derived tumor nucleus, and SCD1 (1:500, Abcam, USA) incubated overnight at 4°C. Immunoreactions were detected with fluorescently labeled secondary antibodies (1:500, Thermo Fisher Scientific, USA). For Bodipy staining, after incubation with the secondary antibody, the cells were incubated with PBS containing Bodipy (1:1000, Thermo Fisher Scientific, USA) for 15 min. Finally, we placed a drop of fluorescence quenching mounting medium containing DAPI on each slide and observed images under a fluorescence microscope (Olympus FV1000, Japan).

### Fluorescence-activated cell sorting

To isolate microglia from mouse brains, we added 4 mL of pre-prepared tissue digestion solution (1 mL RPMI-1640 medium +10 mL DNAse I (10 mg/mL, Sigma-Aldrich, USA) + 20 μL collagenase II (15%, Absin, China) to digest brain tissues in a 37°C incubator for 45 min. The digestion was terminated by adding an equal volume of RPMI-1640 medium containing 10% FBS. After centrifugation at 500 g for 5 min, the supernatant was carefully aspirated. Subsequently, 37% standard isotonic Percoll (SIP, 37% Percoll in HBSS) solution was added to the precipitate and then aspirated into a new tube containing 70% SIP. We then added a 30% SIP solution to each tube. The mixed samples were then centrifuged at 300 g for 40 min. The gray-white cells were transferred to a tube containing pre-cooled HBSS and centrifuged at 500 g for 7 min. After centrifugation, the upper layer of liquid was removed, and the cells were washed with flow cytometry buffer (BD Biosciences, USA). Using FVS780 (1:100, BD Biosciences, USA) to assess cell viability, CD16/CD32(1:100, BD Biosciences, USA) was added for blocking, and the samples were incubated with APC-CD11b (1:200, BioLegend, USA), PerCP/Cyanine5.5-CD45 (1:200, BioLegend, USA) and Bodipy. CD45int CD11b+ microglial cells were collected, and differences in Bodipy 493/503 (1:1000, Thermo Fisher Scientific, USA) content among microglia from different groups were observed. Experimental results were analyzed using FlowJo software.

### RNA extraction and RT-qPCR

For the microglia sorted in the mouse brain, RNA was isolated from the cells using an RNeasy Plus Micro kit (Qiagen, Germany). According to the manufacturer’s instructions, the cells were transferred into 1.5 mL RNase-free tubes containing 75 μL Buffer RLT for lysis. After adding the sample, the vortex was performed immediately to ensure thorough cell lysis, followed by the addition of 75 μL of 70% ethanol to the tubes, whereupon the lysate was transferred onto the spin columns and placed into the provided 2 mL collection tubes. After centrifugation at 8,000 g for 15 s, the supernatant was discarded, and 700 μL of Buffer RW1 was added, followed by centrifugation at 8,000 g for 15 s. Subsequently, 500 μL of Buffer RPE was added, and another centrifugation was performed at 8,000 g for 15 s. After removing the liquid from the tube, 500 μL of 80% ethanol was added to the spin column, followed by centrifugation at 8,000 g for 2 min. The spin column was then placed in a new 1.5 mL RNase-free tube, and 14 μL of RNase-free water was added to the center of the column. After centrifugation at 8,000 g for 1 min, we obtained the RNA dissolved in water. The RNA extraction process from cultured cells was performed with Trizol Reagent (Invitrogen, USA). RNA concentration and purity were measured using a Multi-Detection Microplate reader (Biotek Synergy H1, USA). We performed quantitative real-time PCR on the Real-time fluorescence quantitative PCR instrument (Bio-Rad CFX Connect, USA). Relative expression was normalized relative to the expression of Actin in each sample. The primer sequences are listed in Supplementary Table S1.

### Immunohistochemistry

Human brain paraffin-embedded sections were cut and subjected to deparaffinization with xylene and dehydration with ethanol. The primary antibodies (IBA1, 1:400, Wako, Japan, and SCD1, 1:200, Abcam, USA**)** were applied to the tissue sections, followed by overnight incubation. Then the samples were incubated with HRP-labeled secondary antibodies (1:1000, Jackson ImmunoResearch, USA) for 50 min. Then DAB staining solution (Beyotime Biotechnology, China) was applied to the tissue, with monitoring of staining under a microscope until the sections had turned brownish-yellow. The sections were then immersed in hematoxylin for 5 min to label the cell nucleus. The slides were observed under a microscope (Olympus BX51, Japan).

### Cytokine Array

The Proteome Profiler human cytokine array (R&D Systems, USA) was used to detect 36 cytokines in conditioned media according to the manufacturer’s instructions. The membranes were blocked for 60 min with an array-blocking buffer, then incubated with 1.5 mL of conditioned media overnight at 4 °C. After incubation, the membranes were rinsed three times and treated with streptavidin-HRP for 30 min. Finally, the membranes were examined using imaging equipment (Bio-Rad ChemiDoc XRS + , USA), and spots were quantified using the Image J software program, normalizing each spot to the positive controls.

### Phospho-antibody array

After collecting cells from different treatment groups and adding them to the lysis buffer, we performed phospho-antibody array detection in collaboration with Wayen Biotechnology (Shanghai, China). Here, a phospho-protein microarray (CSP100 plus, Fullmoon Biosystem, USA) was used to screen 16 related signaling pathways, using an array containing 304 antibodies including 157 phosphoproteins, each of which had 6 replicates. We then scanned the array on a SureScan Dx Microarray Scanner (Agilent Technologies, USA) and analyzed the fluorescence intensity by GenePix Pro 6.0. The degree of phosphorylation signal was expressed as the ratio of a protein’s phosphorylated form to its non-phosphorylated form.

### Cell proliferation assay

We used the EdU staining proliferation assay kit (Beyotime Biotechnology, China) to evaluate the proliferation ability of cells. Following the manufacturer’s instructions, cells were cultured with the prepared EdU working solution in a 37 °C incubator for 2 h, followed by fixation with 4% PFA. Cell membranes were permeabilized with PBS containing 1% Triton X-100. Then, we added the reaction solution to the cells for incubation in the dark for 30 min at room temperature. After Hoechst staining of cell nuclei, slides were sealed using an anti-fade solution (Beyotime Biotechnology, China). We then used fluorescence microscopy to capture images and counted the number of cells and the number of EdU-positive cells in each field of view. Six random fields were selected for each slide, and the final results were averaged for statistical analysis.

### Statistical analysis

Data were analyzed and plotted using GraphPad Prism 8, with results presented as mean ± standard error. Each experiment was independently repeated at least three times. In animal experiments, each group consisted of no fewer than 5 mice. When comparing samples between two groups, if the data followed a normal distribution, a paired t-test was used for analysis; if the data did not follow a normal distribution, the Mann-Whitney test was employed. When comparing results among three groups, the obtained data were first subjected to normality testing. If the data followed a normal distribution, one-way ANOVA was used for comparative analysis. P values < 0.05 were considered statistically significant.

## Results

### Microglia surrounding lung cancer brain metastases exhibit increased SCD1 expression and lipid droplet accumulation

**Fig. 1:**
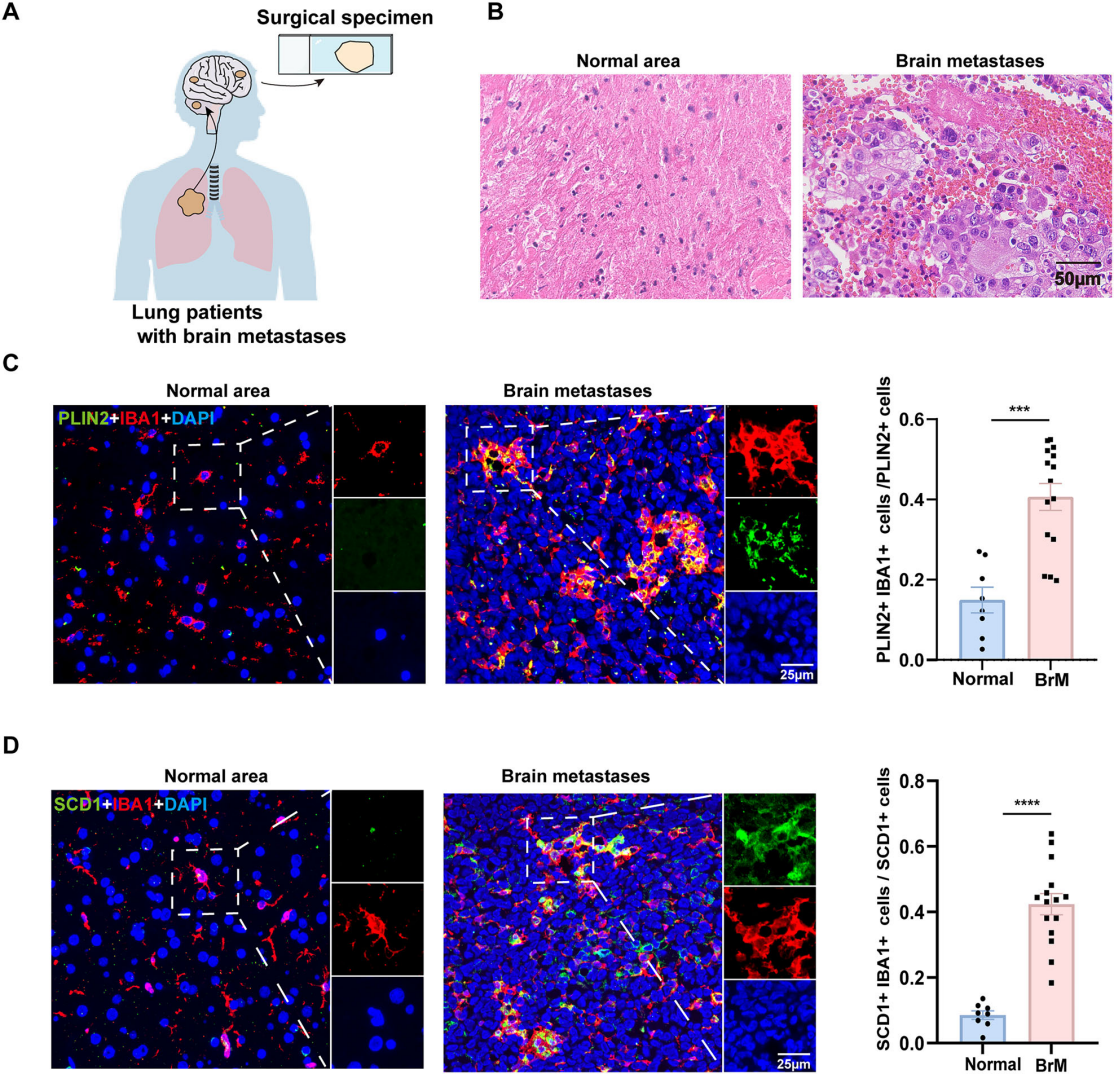
The microglia in the brain metastatic regions of patients exhibit high expression of SCD1 and increased synthesis of lipid droplets. **A** Schematic diagram of intracranial surgical specimens from lung cancer patients with brain metastases. **B** Representative images of brain metastatic tumor sections from lung adenocarcinoma patients stained with HE; the nucleus appears blue, while the cytoplasm and cytoplasmic matrix appear red. Scale bar = 50 μm. **C** Representative immunofluorescence staining images showing the expression of PLIN2 in normal brain lesions and brain metastatic lesions of lung adenocarcinoma patients (n = 15). Scale bar, 25 μm. **D** Representative immunofluorescence staining images showing the expression of SCD1 in normal brain lesions and brain metastatic lesions of lung adenocarcinoma patients (n = 15). Scale bar, 25 μm. (***P < 0.001, ****P < 0.0001).

To explore the impact of tumor cells on the lipid metabolism of microglia in vivo, we first collected paraffin-embedded sections from lung adenocarcinoma brain metastasis patients (Fig. 1A). We used hematoxylin and eosin (HE) staining to determine the formation and location of brain metastatic foci in the specimens. This result revealed numerous cell aggregations featuring larger nuclei and a noticeable presence of mitotic figures, which is indicative of adenocarcinoma-like changes (Fig. 1B). To observe lipid droplet synthesis in microglia within clinical paraffin-embedded sections, we performed co-staining with PLIN2 (Perilipin 2, a key regulatory protein localized on lipid droplet surfaces) and IBA1. We found that microglia in brain metastatic regions exhibited high expression of PLIN2 (Fig. 1C). Furthermore, the expression of SCD1 was also significantly elevated in microglia in tumoral areas compared to normal brains (Fig. 1D). These results suggest that microglia undergo metabolic reprogramming within the brain metastatic microenvironment.

**Fig. 2:**
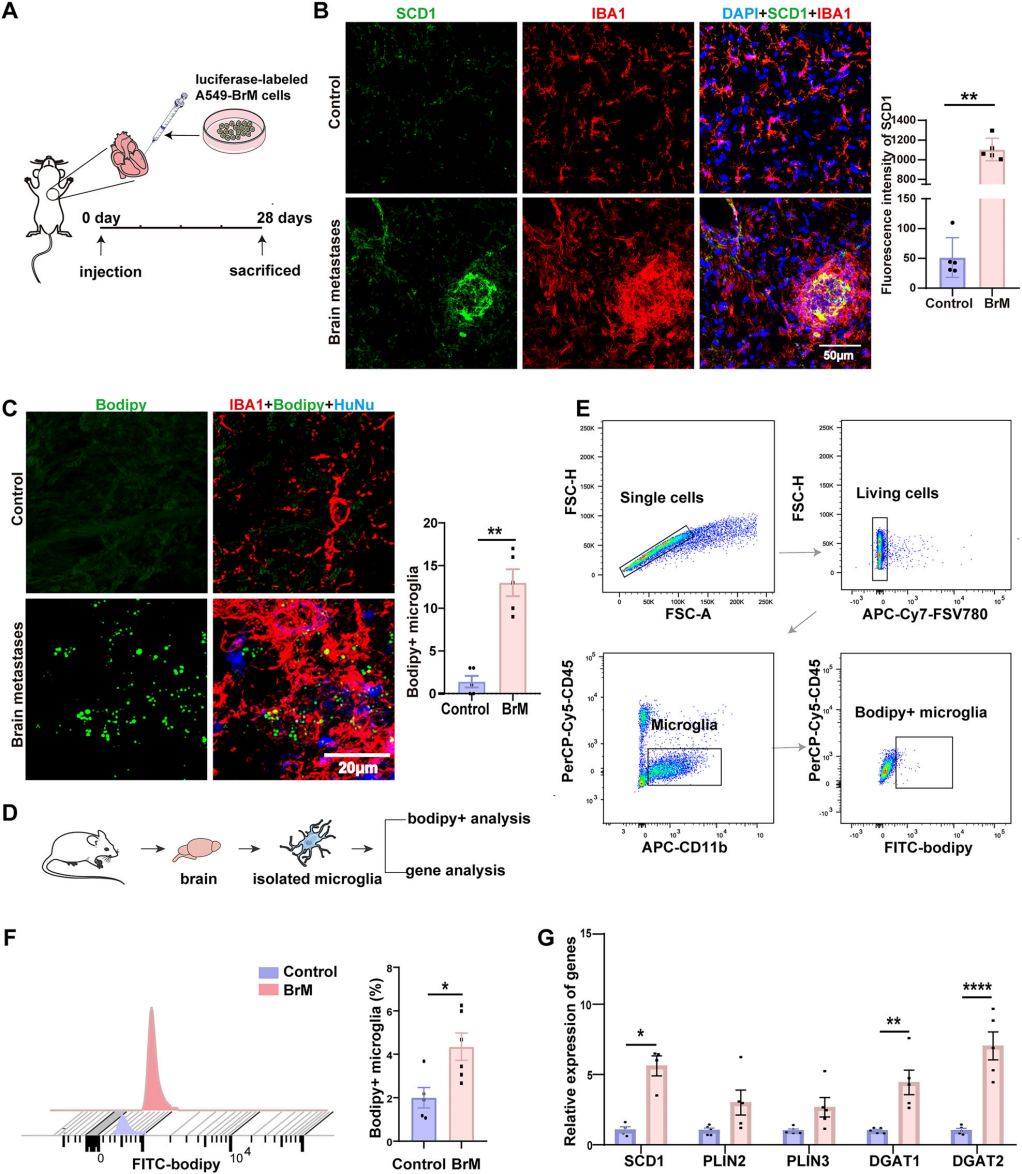
Microglia surrounding lung cancer brain metastases in a mouse model exhibit increased SCD1 expression and lipid droplet accumulation. **A** Schematic diagram illustrating the establishment of a brain metastasis model through lung tumor cell injection into the left ventricle. **B** Representative immunofluorescence staining images showing the expression of SCD1(green) in mouse brain tissues of Control (no tumor cell injection, n = 5) and BrM (brain metastasis model with tumor cell injections, n = 5), IBA1 (red) labeled microglia, and DAPI (blue) marked the cell nuclei (n = 5). Scale bar = 50 μm. **C** Representative images showing HuNu (blue) labeled tumor cell nuclei, Bodipy (green) labeled lipid droplets, and IBA1 (red) marked microglia. Scale bar = 20 μm. Quantification of the number of lipid droplets in microglia per field of view (n = 5). **D** Experimental schematic of the microglia sorting from mouse brain samples. **E** Sorting CD11b^+^ CD45^int^ microglia from mouse brain by flow cytometry. **F** Bodipy+ fluorescence intensity in microglia from mouse brain quantified by flow cytometry analysis. Statistical analysis comparing the percentages of Bodipy+ microglia between brain metastasis and control mice (n = 6). **G** Expression levels of SCD1, PLIN2, PLIN3, DGAT1, and DGAT2 in flow cytometry-isolated microglia by RT-qPCR (n = 5). (*P < 0.05, **P < 0.01, ****P < 0.0001).

Next, we constructed a mouse brain metastasis model. Luciferase-labeled A549-BrM cells were injected into the left ventricle of mice (Fig. 2A), resulting in visible brain tumor signals 4 weeks later. In particular, we observed a significant accumulation of IBA1-positive cells around the brain metastases, and a significantly increased SCD1 fluorescence intensity in brain metastasis mice as compared with the control group (Fig. 2B). Additionally, there were more lipid droplets (Bodipy + ) within the microglia (IBA1 + ) surrounding tumor cells (HuNu + ) (Fig. 2C). To further confirm the changes in microglia lipid metabolism, we used flow cytometry to sort microglia from the mouse brains (Fig. 2D, E). We found increased numbers of Bodipy+ microglia in the brains of brain metastasis mice (Fig. 2F). Then we extracted RNA from the sorted microglia to detect the expression of the SCD1 and lipid droplet synthesis-related genes, including PLIN2, PLIN3, DGAT1, and DGAT2 in the microglia. The results revealed an increased expression of the SCD1 gene in microglia in brain metastasis mice, along with significantly increased expression of DGAT1 and DGAT2 (Fig. 2G). Thus, mice with lung cancer brain metastases have increased expression of SCD1 and lipid droplets in microglia surrounding the lesion.

### Brain metastatic lung cancer cells stimulate microglia to increase their expression of SCD1 and accumulation of lipid droplets

**Fig. 3:**
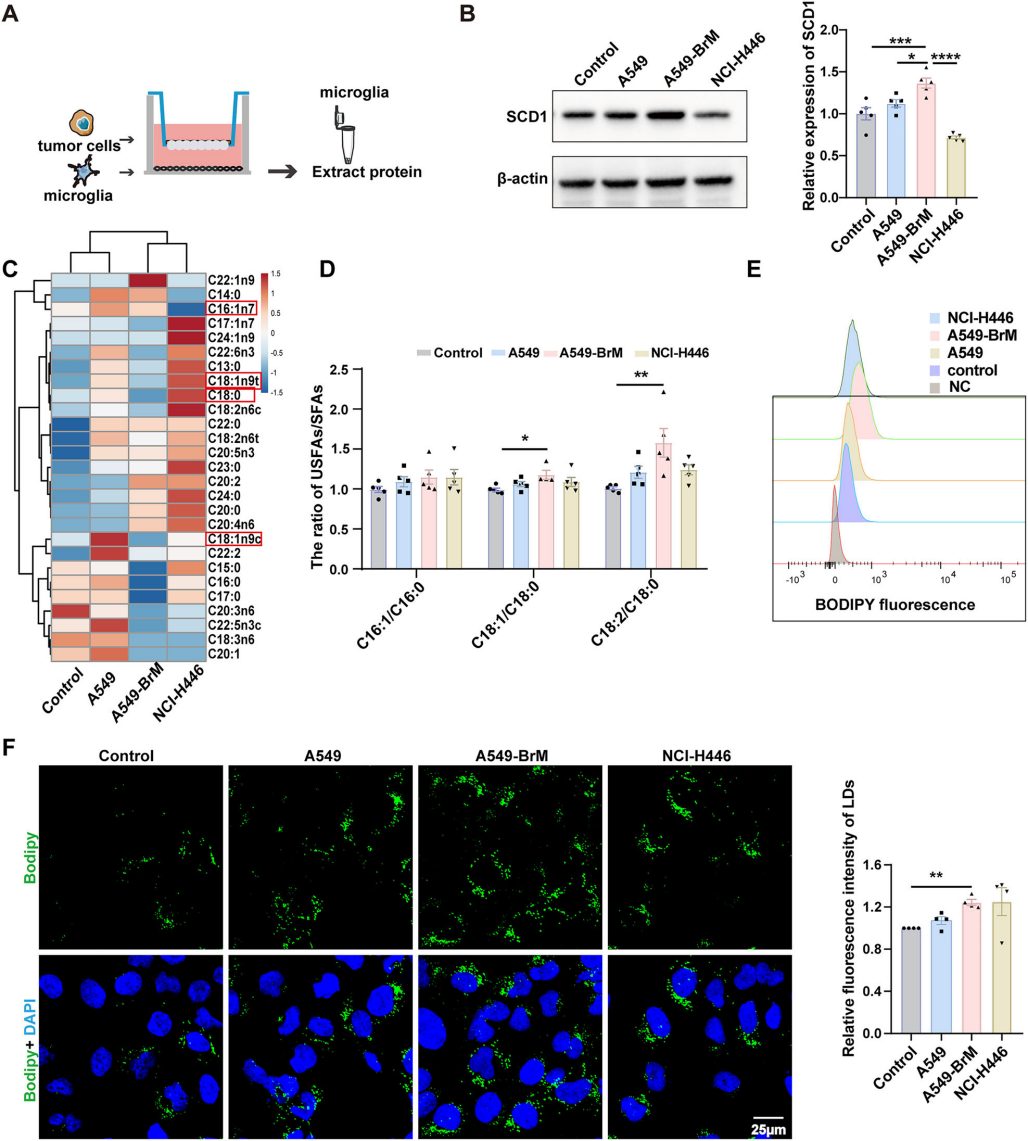
Brain metastatic lung cancer cells stimulate microglia to increase the expression of SCD1 and lipid droplets. **A** Schematic diagram of co-culture experiment between microglia and lung cancer cells. **B** Representative western blot images showing the expression of SCD1 in microglia co-cultured with lung cancer cells or in control conditions. Data were normalized to the control (n = 5). **C** The heatmap showing the concentrations of 27 fatty acids in microglia co-cultured with lung cancer cells or a control group. **D** Statistical chart showing the ratios of unsaturated fatty acids to saturated fatty acids, indicating changes in SCD1 activity (n = 5). **E** The fluorescence intensity of lipid droplets in microglia as detected by flow cytometry. **F** Representative immunofluorescence staining showing lipid droplets (Green) in microglia co-cultured with tumor cells or control, Bodipy (green) labels LDs, and DAPI (blue) marks the cell nuclei. Quantification of the LDs is shown (n = 4). Scale bar =25 μm. (*P < 0.05, **P < 0.01, ***P < 0.001, ****P < 0.0001).

To investigate whether lung cancer cells induce changes in the expression of SCD1 in microglia, we co-cultured microglia HMC3 cells with different lung cancer cells, including A549 lung adenocarcinoma cells, brain metastatic lung adenocarcinoma cells A549-BrM cells, as well as NCI-H446 small cell lung cancer cells (Fig. 3A). After 24 h, we found upregulated SCD1 expression in microglia co-cultured with A549 cells and A549-BrM cells, with the latter group showing the most significant increase (Fig. 3B). As a key enzyme in the fatty acid metabolism process, SCD1 catalyzes the desaturation of fatty acids such as stearic acid (C18:0) and palmitic acid (C16:0) to produce the corresponding mono-unsaturated fatty acids oleic acid (C18:1n9) and palmitoleic acid (C16:1) [27, 38]. Therefore, we used gas chromatography to detect changes in fatty acid content (Fig. 3C). Results showed that A549-BrM brain metastatic lung cancer cells showed significantly increased 18:1n-9/18:0 and 18:2/18:0 ratios in microglia (Fig. 3D). Next, we examined the effect of co-cultured lung cancer cells on lipid droplet formation in microglia, using BODIPY staining to label the lipid droplets. The results showed that A549-BrM cells significantly elevated the number of microglial lipid droplets (Fig. 3E, F). These results indicate that brain metastatic lung cancer cells can promote the expression of SCD1 and the accumulation of lipid droplets in co-cultured microglia.

### Inhibiting SCD1 activity can modulate the responsiveness of microglia to inflammatory stimuli and suppress tumor cell proliferation

**Fig. 4:**
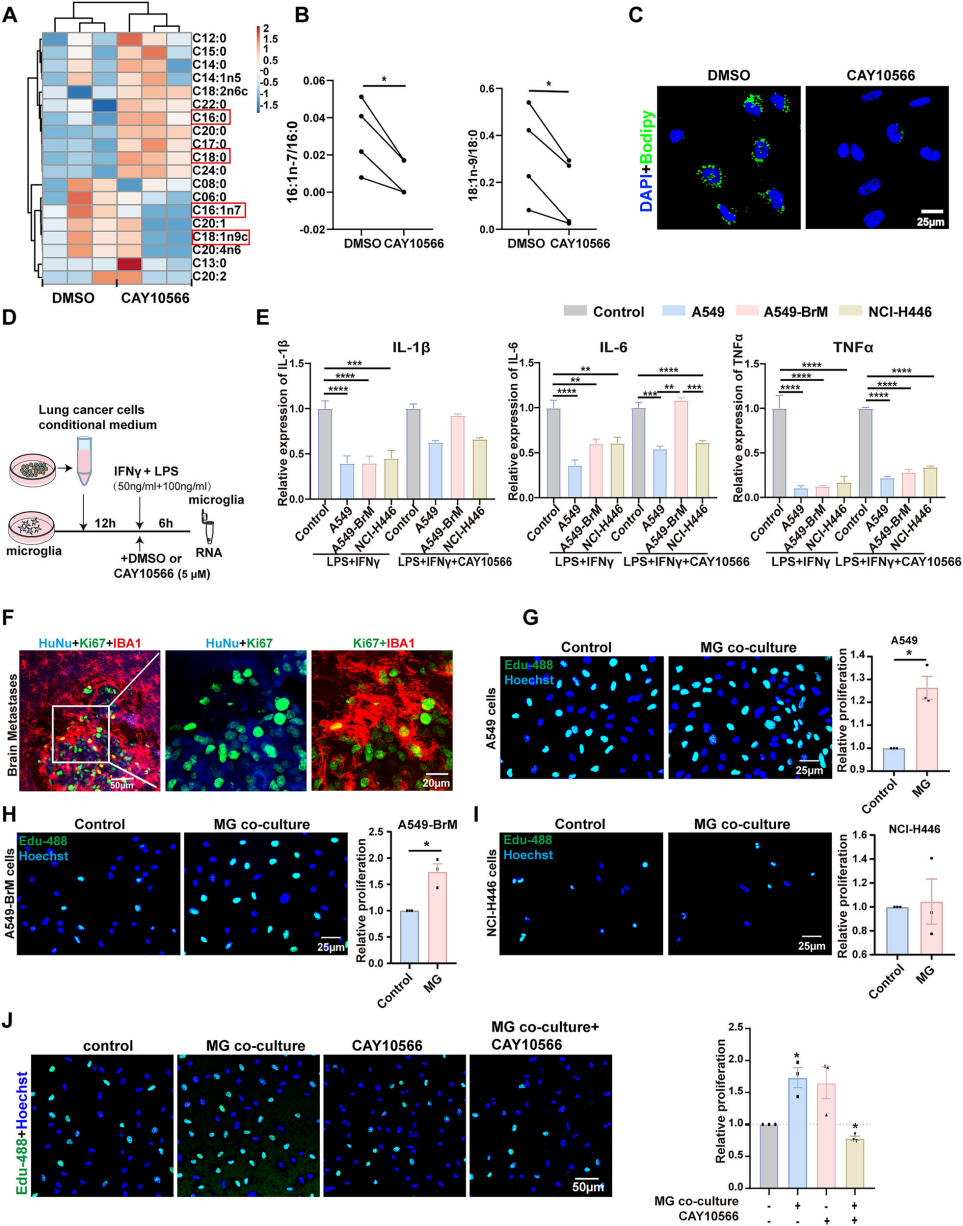
Inhibiting SCD1 activity can modulate the responsiveness of microglia to inflammatory stimuli and suppress tumor cell proliferation. **A** The heatmap showing the fatty acid concentrations detected by gas chromatography in microglia after the addition of 5 μM CAY10566. **B** GC/MS analysis of the SCD1 activity from control (DMSO) and CAY10566 (5 μM) treatment group; desaturation indices were determined by calculating the 16:1/16:0 and 18:1/18:0 ratios (*n* = 4). **C** Representative images showing that CAY10566 treatment in vitro can reduce lipid droplet formation in microglia. Bodipy (green) labels LDs, and DAPI (blue) marks the cell nuclei. Scale bar, 25 μm (*n* = 4). **D** Schematic diagram illustrating the experiment for investigating microglial inflammatory response. **E** Microglia were preconditioned with conditioned media from lung cancers for 12 h before 6 h stimulation with LPS (100 ng/mL) and IFN-γ (50 ng/mL). Expression levels of IL1β, IL6, and TNFα in microglia pre-treated with tumor-conditional medium or control were detected by RT-qPCR (n = 4). **F** Representative immunofluorescence staining image showing the proliferation of tumor cells in the mouse brains, with (blue) HuNu-labeled nuclei of tumor cells, (red) IBA1-labeled microglia, and (green) Ki67-labeled proliferating cells. Scale bar = 20 μm. **G** Representative EDU staining images showing the proliferation capacity of A549 cells co-cultured with microglia or the control group (n = 3). **H** Representative EDU staining images showing the proliferation capacity of A549-BrM cells co-cultured with microglia or the control group (n = 3). **I** Representative EDU staining images showing the proliferation capacity of NCI-H446 cells co-cultured with microglia or the control group (n = 3). **J** Representative EDU staining images showing the proliferation capacity of A549-BrM cells co-cultured with microglia treated with or without SCD1 inhibitors (n = 3). (*P < 0.05, **P < 0.01, ***P < 0.001, ****P < 0.0001).

To observe the effects of altered SCD1 activity on microglial lipid metabolism, we applied the SCD1 inhibitor CAY10566 and used gas chromatography to detect the fatty acid content (Fig. 4A). The ratios of 16:1n7/16:0 and 18:1n-9/18:0 showed a significant reduction in microglia after CAY10566 treatment (5 μM) compared to the control group (treated with solvent DMSO alone without drugs) (Fig. 4B). Additionally, Bodipy staining revealed a significant decrease in lipid droplet abundance after the treatment (Fig. 4C). These results demonstrated that the SCD1 inhibitor CAY10566 could effectively suppress SCD1 activity and attenuate intracellular lipid droplet accumulation.

Next, we explored the impact of SCD1 activity on microglial function. Previous research has revealed a strong correlation between lipid alterations and the inflammatory status of cells, whereby lipids can influence the activation state of tumor-associated macrophages in response to inflammation [35, 39, 40]. In an initial test of the association between SCD1 activity and microglial function, we found that CAY10566 had no significant effect on microglial inflammatory status (Figure. S1A, B). Subsequently, we induced a pro-inflammatory state in microglia using the well-established pro-inflammatory factors LPS and IFN-γ. Then we assessed the responsiveness of microglia to pro-inflammatory stimuli under conditions that mimic a tumor environment by adding conditional medium from lung cancer cells (Fig. 4D). Interestingly, administration of LPS and IFN-γ after pre-treatment with a tumor-conditioned medium from A549, A549-BrM, or NCI-H446 cells, there was a significant decrease in the levels of cytokines including IL-1β, IL-6, and TNFα. Interestingly, pharmacological inhibition of SCD1 using CAY10566 selectively increased the expression of IL-1β and IL-6 in microglia exposed to A549-BrM conditioned medium, while showing no significant effects in other treatment groups (Fig. 4E). These findings suggest that A549-BrM cells can diminish the reactivity of microglia to inflammatory stimuli, but that blockade of SCD1 activity with CAY10566 can restore the inflammatory response of microglia.

In the brains of mice with brain metastases, we observed a significant proliferation of tumor cells (Fig. 4F). To investigate further the impact of microglia on the proliferation of tumor cells, we initially examined changes in the proliferative capacity of tumor cells co-cultured with microglia in vitro. We found that microglia could enhance the proliferation ability of A549 and A549-BrM cells (Fig. 4G–I). Moreover, there was a clear promotion of A549-BrM cell proliferation in the co-culture system, and the direct addition of CAY10566 to the culture medium did not significantly affect their proliferation ability. However, the addition of CAY10566 to inhibit SCD1 activity in microglia during co-culturing with tumor cells significantly reduced the promoting effect of microglia on tumor cell proliferation and even inhibited tumor growth (Fig. 4J). These results indicate that pharmacological inhibition of SCD1 activity in microglia reduces their promoting effects on tumor proliferation.

### Administration of an SCD1 inhibitor in conjunction with a CSF1R inhibitor for the treatment of lung cancer brain metastases

**Fig. 5:**
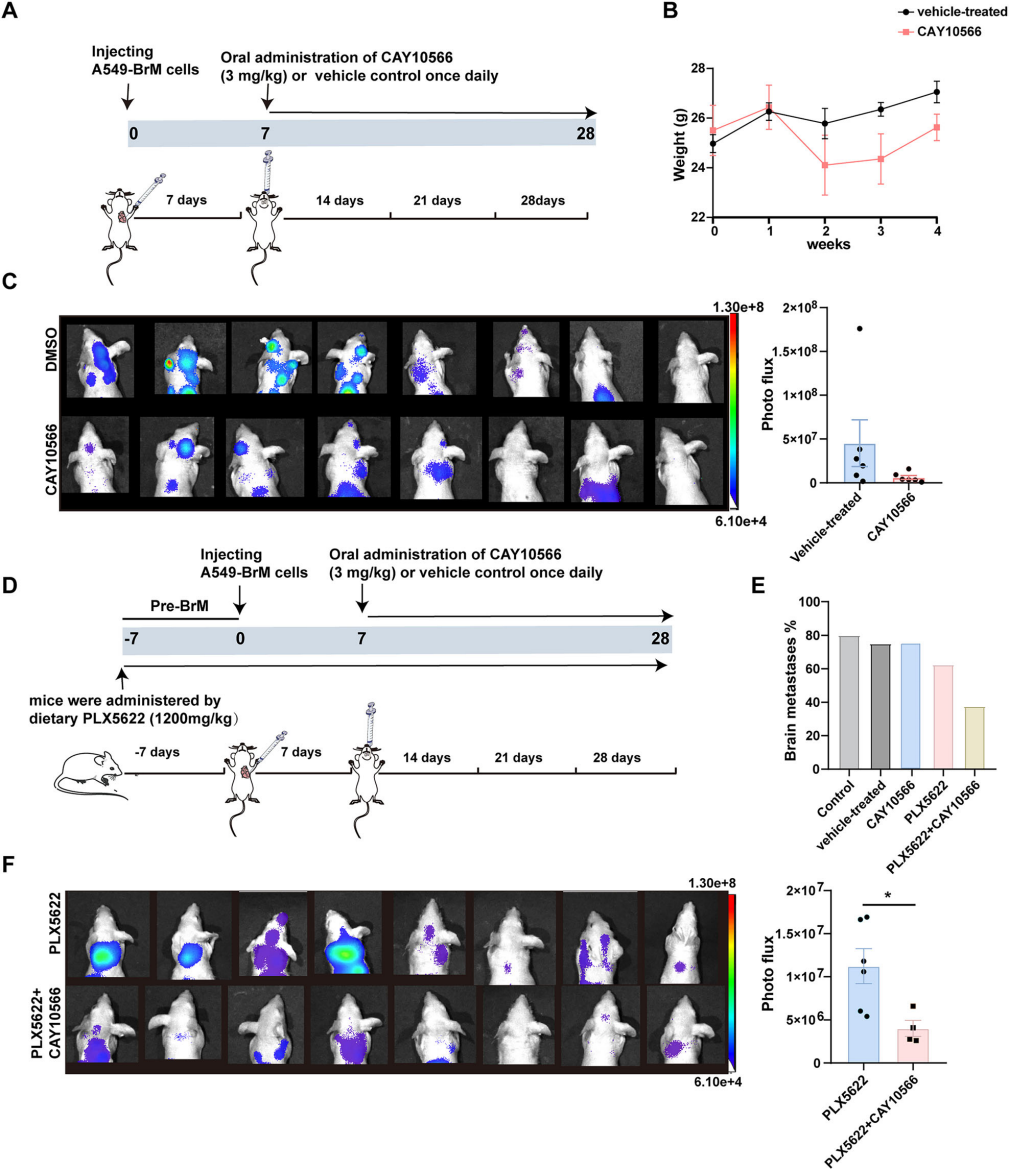
The administration of an SCD1 inhibitor in conjunction with a CSF1R inhibitor in mice with brain metastases. **A** Schematic diagram of experimental design (**B**, **C**). **B** Body weight changes in mice during treatment with CAY10566 (3 mg/kg, dissolved in DMSO and diluted in corn oil) or vehicle (only DMSO diluted in corn oil) (n = 8). C. Bioluminescence imaging and quantification of brain metastases burden in vehicle-treated and CAY10566 groups (n = 8). **D** Schematic for experimental design (**E**, **F**). **E** The incidence of brain metastasis in the control group (untreated model), vehicle-treated group, CAY10566 group, PLX5622 group, and the combination treatment group of PLX5622 + CAY10566 after tumor cell inoculation (n = 8). **F** Bioluminescence imaging and quantification of brain metastases burden in PLX5622 and PLX5622 + CAY10566 groups. (*P < 0.05).

To investigate the impact of the SCD1 inhibitor on brain metastases in vivo, we injected A549-BrM cells into the left cardiac ventricle of mice and initiated oral administration of CAY10566 (3 mg/kg) one week later. The vehicle-treated group received an equal volume of the vehicle. Starting from the fourth week, tumor formation and size were monitored in mouse brains (Fig. 5A). The treatment with CAY10566 caused an initial decrease in body weight, followed by recovery after one week of treatment (Fig. 5B). Tumor formation persisted in the brains of mice receiving inhibitor treatment, without any significant alteration in the rate of brain metastasis formation. However, measurement of metastatic tumor size demonstrated a non-significant decreasing trend in mice treated with the oral SCD1 inhibitor compared to the control group (Fig. 5C). Results showed that alterations in SCD1 activity in microglia influenced their responsiveness to inflammatory stimulation, leading to reduced reactivity towards inflammatory stimuli and promotion of tumor cell proliferation. However, the administration of SCD1 inhibitors was ineffective in inhibiting pre-existing brain tumors. Therefore, we hypothesized that SCD1 inhibitor administration in vivo might yet enhance their susceptibility to other immunotherapeutic agents and overcome therapy resistance. To test this, we applied inhibitors of Colony Stimulating Factor 1 Receptor (CSF1R) in combination with the SCD1 inhibitor to evaluate their therapeutic efficacy against brain metastases. The CSF1-CSF1R pathway plays a crucial role in the proliferation and differentiation of microglia/macrophages, and the orally administered CSF1R inhibitor PLX5622 exhibits excellent blood-brain barrier penetration, with minimal adverse effects, ensuring a high level of safety [41, 42]. Nonetheless, clinical trials with PLX5622 have not yielded satisfactory outcomes, possibly due to the emergence of drug resistance [43–45]. Therefore, we proposed combining CSF1R inhibition with other treatment modalities as a promising novel therapeutic strategy. Thus, we investigated the combined administration of CSF1R and SCD1 inhibitors to assess their combined impact on tumor growth in a mouse model of brain metastasis. Mice were fed with PLX5622 for one week before the tumor cell injection, with initiation of daily CAY10566 oral administration after one week. We tracked brain tumor formation for four weeks (Fig. 5D), finding that oral PLX5622 alone reduced the formation of brain metastases (60%) compared to the control group and the oral CAY10566 alone treatment (70% ~ 80%). The combination of PLX5622 and CAY10566 significantly decreased the formation rate of brain metastases (40%) (Fig. 5E). Living imaging analysis demonstrated a significant reduction in tumor sizes among mice receiving combination therapy (Fig. 5F).

### Lung cancer cells stimulate the activation of the STAT3 signaling pathway in microglia to promote the expression of SCD1

**Fig. 6:**
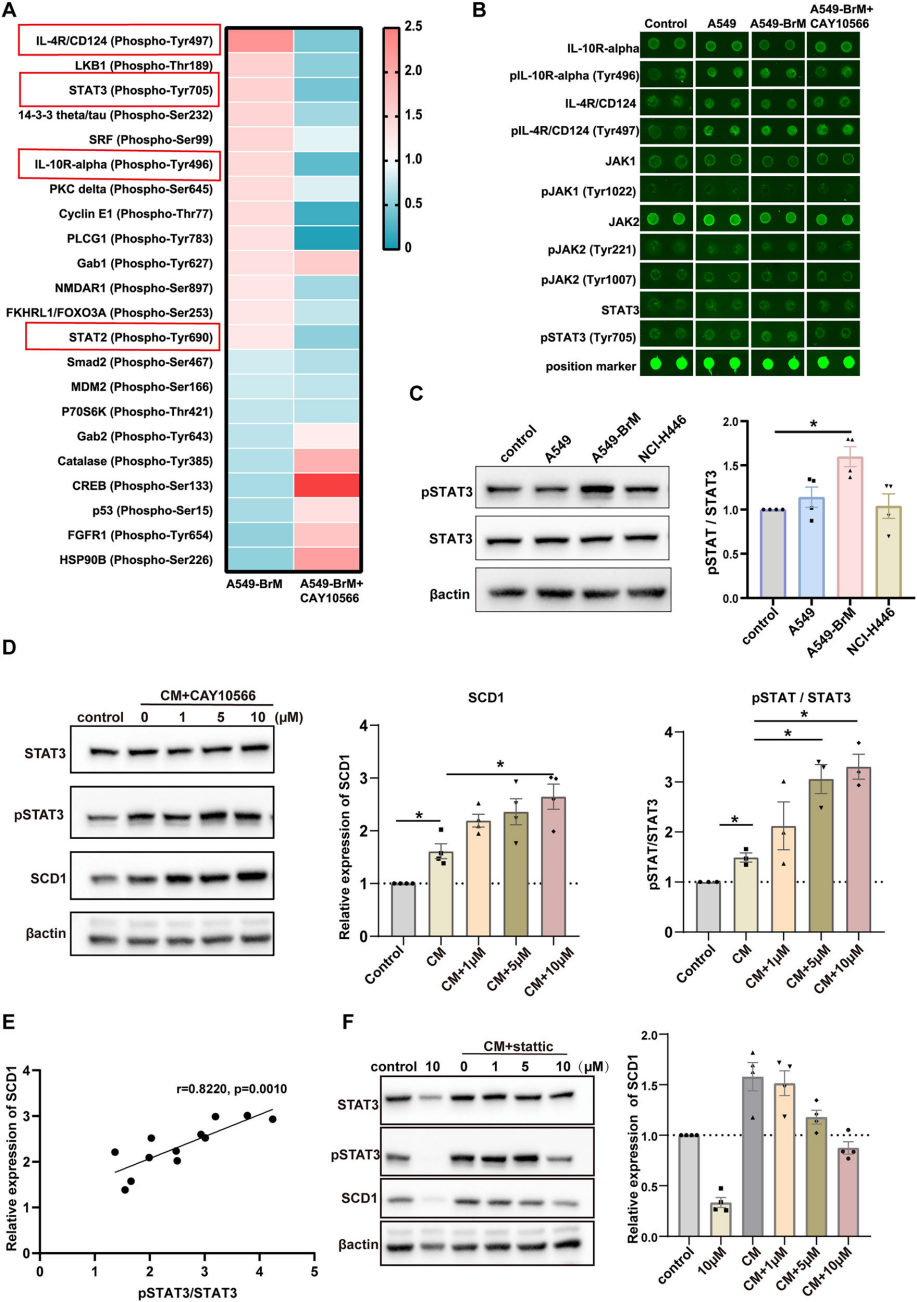
Lung cancer cells stimulate the activation of the STAT3 signaling pathway in microglia to promote the expression of SCD1. **A** Heatmap showing significant phosphorylated protein differences between the CAY10566-treated group and the untreated group in microglia pretreated with A549-BrM medium. **B** Phospho-proteins of the JAK-STAT3 signaling pathway in the phospho-antibody array. **C** Representative western blot images showing the expression of pSTAT3 and STAT3 in microglia co-cultured with lung cancer cells or control, and the histogram showing the quantitative array of the ratio of pSTAT3 to STAT3 (n = 4). **D** Western blot results showing the expression of SCD1, STAT3, and pSTAT3 in microglia cultured in the presence of different concentrations of CAY10566 (n = 4). **E** Correlation analysis showing a positive correlation between the expression of SCD1 and pSTAT3/STAT3. **F** Western blot results showing the expression of SCD1, STAT3, and pSTAT3 in microglia cultured in the presence of different concentrations of Stattic (n = 4). (*P < 0.05).

To investigate the molecular mechanisms associated with SCD1 changes in microglia, we used a phosphorylated protein chip to screen for relevant signaling pathways. Considering earlier findings of pronounced microglial lipid changes in A549-BrM brain metastatic lung cancer cells, we compared the alterations in cell signaling pathways among three groups of microglia by co-culturing A549-BrM brain metastatic lung cancer cells or normal A549 cells. We detected intricate interactions between the tumor cells and microglia involving the activation of multiple signaling pathways in microglia, including the MAPK, JAK-STAT, NF-κB, and mTOR pathways (Figure. S2A-D). To determine the specific signaling pathway closely associated with altered SCD1 expression in microglia, we added CAY10566 to the A549-BrM conditioned medium and compared the phosphorylated proteins related to significant pathway differences between the CAY10566-treated and untreated groups. The JAK-STAT3 signaling pathway was predominantly activated in the microglia of the A549-BrM group, but its activation was attenuated by CAY10566 (Fig. 6A, B). Further analysis of cellular proteins revealed a pronounced STAT3 activation specifically in microglia treated with A549-BrM conditioned medium (Fig. 6C). Thus, the alteration of SCD1 expression in microglia, induced by brain metastatic cancer cells, was linked to the activation of the STAT3 signaling pathway.

Subsequently, we examined the impact of different concentrations of CAY10566 on SCD1 gene expression and STAT3 phosphorylation levels in microglia. Interestingly, higher CAY10566 concentration was associated with enhanced negative feedback regulation of SCD1 protein expression, accompanied by elevated STAT3 phosphorylation (Fig. 6D). Notably, we saw a positive correlation between these two factors (Fig. 6E). We next introduced Stattic (the inhibitor of the STAT3 signaling pathway) into the culture medium. Stattic reduced STAT3 phosphorylation and importantly led to a downward trend in SCD1 protein expression (Fig. 6F). These findings suggest that inhibition of the STAT3 signaling pathway suppresses SCD1 protein expression. Combining these results with previous experimental data indicates that A549-BrM cells promote activation of the STAT3 signaling pathway in microglia and enhance the expression of SCD1 protein.

## Discussion

In this study, we found that brain metastatic lung cancer cells can promote the expression of SCD1 in microglia, leading to alterations in the fatty acid composition and increased accumulation of lipid droplets. Results suggest that increased SCD1 activity in microglia alters their response to inflammatory stimuli. Treatment with an SCD1 inhibitor ameliorated the inflammatory response of microglia and reduced their pro-proliferative impact on tumor cells. Furthermore, the combined SCD1 and CSF1R inhibitor treatments alleviated brain metastatic tumor burden in mice. Additionally, our study revealed that tumor cells could activate the STAT3 signaling pathway in microglia to promote the expression of SCD1. These findings reveal an altered STAT3-SCD1-lipid metabolism-inflammatory response pathway in microglia in the lung cancer brain metastatic microenvironment and present a potential new strategy for treating brain metastases.

In the tumor microenvironment, lipid metabolism plays a unique and intricate role in tumor occurrence, metastasis, and modulation of tumor immunity [26, 46]. Lipids in the tumor microenvironment exert a rich variety of functions, either promoting tumor growth or impeding tumor progression [25]. Fatty acid metabolism is necessary for maintaining cellular membrane composition and regulating various cellular functions involved in oncogenic signaling pathways, such as endoplasmic reticulum stress signaling and the Wnt/β-catenin pathway [29, 30]. SCD1 as a key enzyme in fatty acid metabolism plays a significant role in cancer cell growth [29, 30, 47]. For instance, some studies have reported that SCD1 inhibitors can diminish the characteristics of cancer stem cells and hinder the growth of cancer cells [48–50]. However, there has been limited investigation into its role in regulating immune cells, and the immune responses against tumors. One study revealed that SCD1-dependent lipid droplet accumulation in cancer-associated fibroblasts would promote the progression of lung cancer [51]. Another recent study showed that the administration of an SCD1 inhibitor in mouse tumor models could directly stimulate dendritic cells and CD8 + T cells to enhance their functions [52]. However, there are hitherto no reports on the role of SCD1 in tumor-associated macrophages (TAMs). For the first time, we observed an increase in SCD1 expression in microglia in the brain metastasis microenvironment and explored the impact of SCD1 upregulation on microglia.

SCD1 can promote the formation of intracellular lipid droplets and regulate their size and lipid composition [33, 34]. Lipid droplets are increasingly recognized as dynamic participants in lipid metabolism and play important roles in immune regulation as well [39]. Enrichment of lipid droplets in TAMs of peripheral tumors can influence mitochondrial respiration, inducing them to differentiate into an immunosuppressive macrophage phenotype. Pharmacological inhibitors of lipid droplet formation disrupted that polarization [36], making SCD1 activity an effective target for inhibiting tumor growth. In central nervous system tumors, accumulation of lipid droplets in TAMs can provoke abnormalities in their cytokine secretion and phagocytic function [53], but little is currently known about the formation and function of lipid droplets in microglia/macrophages in brain tumors. In the present study, we observed an increase in SCD1 expression in tumor-associated microglia, accompanied by lipid droplet accumulation.

In peripheral tumors, long-chain fatty acids (especially unsaturated fatty acids like oleic acid) can induce a reprogramming of TAMs towards an immunosuppressive phenotype [36]. Lipid droplets have been identified as essential organelles [39], and inhibitors of lipid droplet formation have been found to effectively block the polarization of TAMs in vitro, and thus inhibit tumor growth in vivo [36]. Conversely, abnormal lipid flux also directs TAMs towards a tumor-promoting phenotype induced by IL-4, while reducing the anti-tumor response induced by IFN-γ, thereby exerting a pro-tumorigenic effect [54]. Here, we investigated similar phenomena in microglia in the brain tumor microenvironment. Upon application of SCD1 inhibitors to alter lipid metabolism, we observed improved responsiveness of microglia cells to pro-inflammatory stimuli such as LPS and IFN-γ. This indicates that brain metastatic lung cancer cells interfere with anti-tumor responses by reprogramming microglial lipid metabolism, thus promoting pro-tumor polarization.

SCD1 has high expression in various types of cancers and plays a crucial role in cancer cell growth by controlling fatty acid metabolism, thus presenting a potential therapeutic target for cancer [29, 30, 47]. Applying SCD1 inhibitors in mouse colon cancer models can increase the production of CCL4 in CD8 + T cells, moreover, the combination of SCD1 inhibitors and anti-PD-1 antibodies exhibits antitumor effects [52]. Our findings show that the SCD1 inhibitor alone did not affect tumor proliferation in vitro, nor did oral treatment significantly alter the growth of mouse brain metastatic tumors in vivo. These negative results may reflect the complex internal environment of the mouse brain and compensatory metabolic regulation. However, the addition of SCD1 inhibitor to co-cultures of tumor cells and microglia significantly reduced the ability of microglia to promote tumor cell proliferation. This result leads us to speculate that the combination of SCD1 inhibitors with drugs targeting microglia could enhance antineoplastic drug efficacy and overcome resistance.

CSF1-CSF1R, as a crucial pathway for microglial/macrophage proliferation and differentiation, has been targeted in many preclinical brain tumor studies using antibodies or small molecule inhibitors to suppress CSF1R, thereby reducing macrophage infiltration or depleting resident cells that promote tumor microglia/macrophages [41, 43]. Inhibition of CSF1R results in the loss of M2-type markers, suggesting at least partial reprogramming of TAMs towards an anti-tumor M1 phenotype [41, 55]. Experimental CSF1R inhibitors can be orally administered across the blood-brain barrier, without exerting significant side effects [56]. In a mouse glioma model, the application of CSF1R inhibitors can prolong survival or even inhibit tumors, accompanied by the transition of TAMs to an anti-tumor phenotype [44]. In murine breast cancer and melanoma brain metastasis models, CSF1R inhibitors could reduce tumor progression [57, 58]. However, clinical trials of CSF1R inhibitors in glioblastoma have not shown significant effects [42]. Meanwhile, in animal brain metastasis models, the efficacy of CSF1R blockers diminishes with tumor progression or even leads to tumor rebound due to drug resistance [44, 57]. Therefore, combining CSF1R with other treatments may present a new strategy. In a breast cancer brain metastasis model, combining CSF1R and STAT5 inhibitors effectively sustained tumor suppression, while normalizing the phenotypes of TAMs [57]. In our study, we investigated the effect of combining CSF1R and SCD1 inhibitors on the growth of mouse brain metastases. Results showed that the drug combination indeed significantly reduced the formation of brain metastases and decreased tumor burden, thus supporting this approach as a promising translational strategy for treating brain metastases.

We observed a significant activation of the STAT3 signaling pathway in microglia by brain metastases lung cancer cells, resulting in the increased expression of SCD1 protein. In a study on lung cancer brain metastasis, single-cell sequencing revealed that, compared to healthy control microglia, TAMs had increased expression of genes in the JAK/STAT signaling pathway [22]. Genes in that pathway overlapped with present findings of the pathways associated with altered microglial lipid changes in microglial cells. Furthermore, the JAK/STAT study demonstrated IL6 to be the most prominent upstream cytokine influencing the pathway and confirmed an interaction between the IL-6/JAK2/STAT3 signaling pathway in the brain metastatic microenvironment involving lung cancer cells and microglia [22]. Another investigation on lung cancer brain metastasis observed that the nACh receptor-STAT3 pathway mediated a nicotine-induced phenotypic transformation in microglia. Inhibiting STAT3 could block the M2-type transformation of microglia, inhibit the secretion of pro-tumor growth factors, and reactivate their phagocytic activity against tumor cells [59]. These results suggested that STAT3 signaling pathway alterations within tumor-associated microglial cells relate closely to their functional changes. There are currently no studies linking the STAT3 signaling pathway with lipid metabolism in microglia of brain metastases. However, in a recent study, there was significantly up-regulated SCD1 expression in airway epithelial cells of STAT3-knockout mice [60]. Taken together, our experimental results indicate a functional role for the STAT3-SCD1-lipid metabolism axis in microglia cells within the brain metastasis microenvironment.

In conclusion, our findings indicate that lung cancer cells can upregulate the expression of SCD1 in microglia by activating the STAT3 pathway to change microglial inflammatory response, thereby promoting tumor growth in lung-to-brain metastases. Importantly, we find that combined treatment with an SCD1 inhibitor and a CSF1R inhibitor alleviates tumor progression in mice. Nonetheless, we note certain limitations in our study. We have not established the precise molecular mechanisms whereby SCD1 activity influences microglial responsiveness to pro-inflammatory stimuli and modification of tumor cell proliferation. In the murine treatment model, observed effects of SCD1 inhibitors on body weight may have impacted treatment efficacy. While our current therapeutic strategy focuses on the combined inhibition of SCD1 and CSF1R, emerging evidence suggests that other pathways, such as CD47-SIRPα axis blockade, PD-1/PD-L1 immune checkpoint inhibition, may synergize with lipid metabolic reprogramming to enhance therapeutic efficacy [52, 61]. Future investigations will employ microglia-specific SCD1 knockout models coupled with multi-omics profiling to systematically clarify tumor-associated macrophage functional heterogeneity. To make the treatment safer with fewer side effects, our future studies will focus on creating improved dosing plans for SCD1 inhibitors that both fight tumors effectively and keep a healthy metabolism. We’ll also use real-time tracking of blood oleate levels to adjust doses as needed, helping prevent serious metabolic issues while maintaining strong cancer-fighting effects. Despite these limitations, our study finds the alterations in the microglial STAT3-SCD1-lipid metabolism-inflammatory response pathway induced by tumor cells and presents a novel therapeutic strategy for treating brain metastasis.

## Supplementary information

Figure S1

Figure S2

Table S1

Figure legends to the the supplementary figures

western blot original images

## Data Availability

The data analyzed in this research are included in this published article and the supplementary materials. Additional supporting data can be available from the first author upon reasonable request.
