# Supplementary figures and images for: Lung cancer cells upregulate stearoyl-CoA desaturase 1 in microglia by activating the STAT3 pathway to change microglial inflammatory response in lung-to-brain metastases
Source: Cell Death Dis. 2025 Oct 6;16(1):702. doi: 10.1038/s41419-025-08003-2 (PMC12500914; doi:10.1038/s41419-025-08003-2)

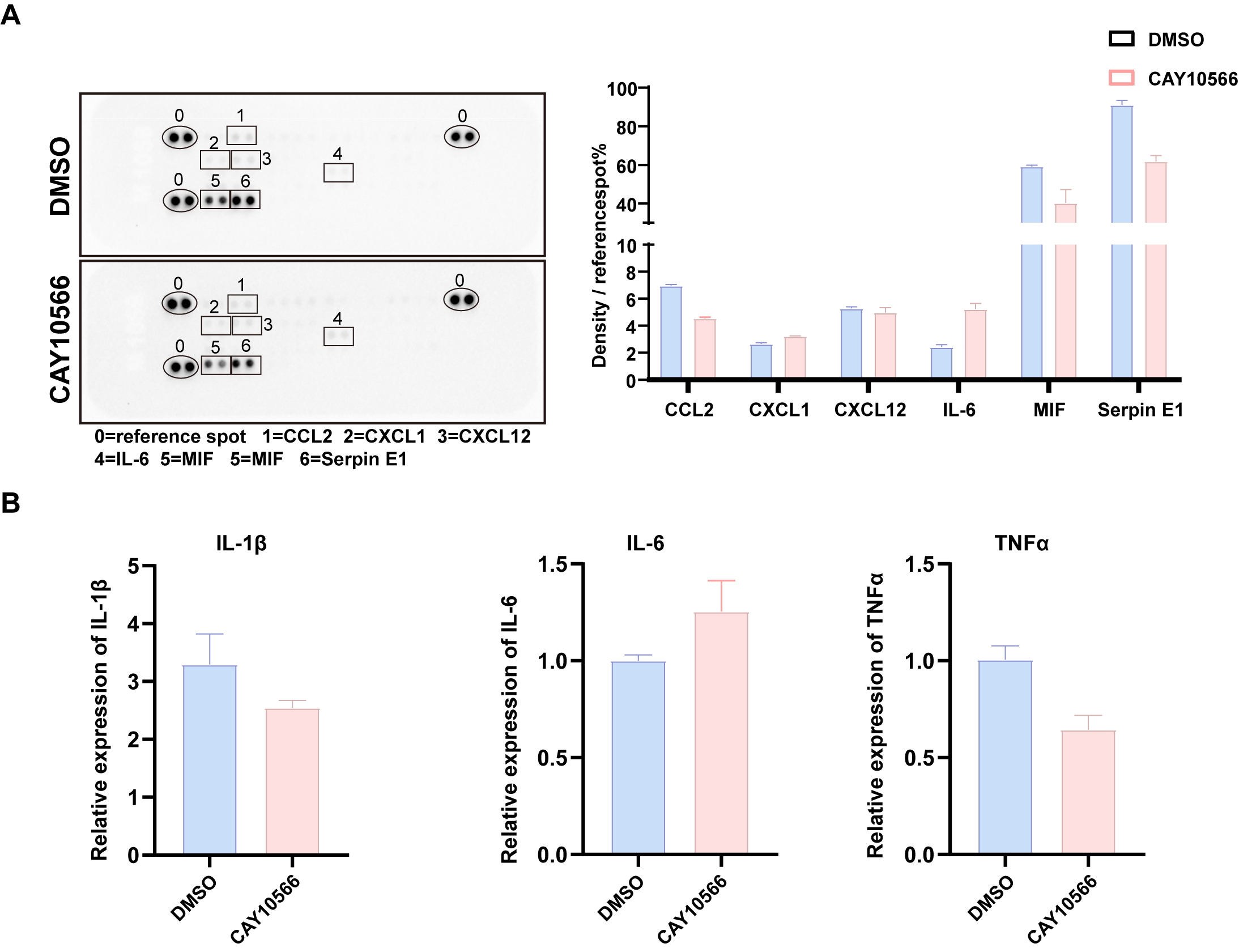

Supplement: Supplementary file 1 — Figure S1 [file 41419_2025_8003_MOESM1_ESM.tif]

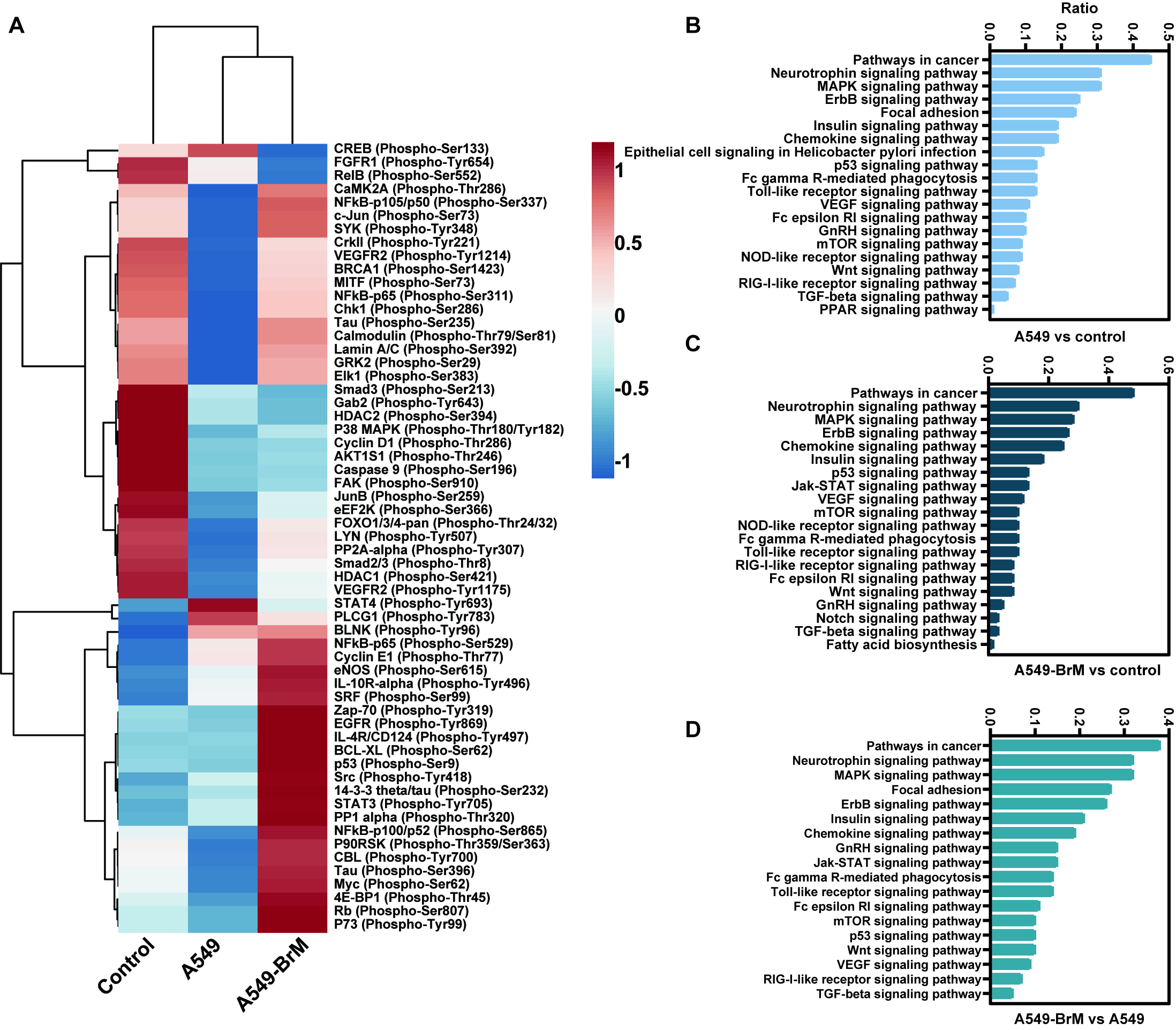

Supplement: Supplementary file 2 — Figure S2 [file 41419_2025_8003_MOESM2_ESM.tif]
