# Supplementary material for: Lung cancer cells upregulate stearoyl-CoA desaturase 1 in microglia by activating the STAT3 pathway to change microglial inflammatory response in lung-to-brain metastases
Source: Cell Death Dis. 2025 Oct 6;16(1):702. doi: 10.1038/s41419-025-08003-2 (PMC12500914; doi:10.1038/s41419-025-08003-2)
Supplement: Supplementary file 3 — Table S1 [file 41419_2025_8003_MOESM3_ESM.docx]

**Table S1.** **Primers for RT-qPCR**

| Gene name | Forward primer | Reverse primer |
| --- | --- | --- |
| hSCD1 | AGCTCCTATACCACCACCACCAC | GGCATCGTCTCCAACTTATCTCCTC |
| mScd1 | AGCCTGTTCGTTAGCACCTTCTTG | GCACCCAGGGAAACCAGGATATTC |
| ABCA1 | GCTCAGTGGGATGGATGGCAAAG | GCTCAGTGGGATGGATGGCAAAG |
| PLIN2 | GCACCCAGGGAAACCAGGATATTC | CTGACATAAGCGGAGGACACAAGG |
| PLIN3 | GCAGCGACAGGAGCAGAACTAC | GCTTGCCGAGGGAGTGTTCATAG |
| DGAT1 | AGCTATCCAGACAACCTGACCTACC | TCAAGAACTCGTCGTAGCAGAAAGC |
| DGAT2 | TCAAGAACTCGTCGTAGCAGAAAGC | TCAGTTCACCTCCAGCACCTCAG |
| IL-1β | GGACAGGATATGGAGCAACAAGTGG | GGACAGGATATGGAGCAACAAGTGG |
| IL-6 | GACAGCCACTCACCTCTTCAGAAC | GCCTCTTTGCTGCTTTCACACATG |
| TNFα | AGCCCTGGTATGAGCCCATCTATC | TCCCAAAGTAGACCTGCCCAGAC |
| hACTB | CCACGAAACTACCTTCAACTCCATC | AGTGATCTCCTTCTGCATCCTGTC |
| mActb | GCTCTCCCTCACGCCATCC | GTCACGCACGATTTCCCTCTC |
