# Supplementary material for: Lung cancer cells upregulate stearoyl-CoA desaturase 1 in microglia by activating the STAT3 pathway to change microglial inflammatory response in lung-to-brain metastases
Source: Cell Death Dis. 2025 Oct 6;16(1):702. doi: 10.1038/s41419-025-08003-2 (PMC12500914; doi:10.1038/s41419-025-08003-2)
Supplement: Supplementary file 4 — Figure legends to the the supplementary figures [file 41419_2025_8003_MOESM4_ESM.docx]

**Fig. S1 CAY10566 had no significant effect on the expression of microglial inflammatory factors**

A. The expressions of 36 cytokines in the supernatants of microglia treated with DMSO or CAY10566 were assessed by a human cytokine array, and the histogram showing the statistical results.

B. Expression levels of IL1β, IL6, and TNFα in microglia treated with DMSO or CAY10566 via RT-qPCR (n = 3).

**Fig. S2 The activation of multiple signaling pathways in microglia co-cultured with lung cancer cells.**

A. Heatmap of phosphorylated proteins in different groups.

B. GO enrichment analysis showing the phosphorylated proteins differentially expressed in microglia from A549 and control group are involved in various cellular pathways.

C. GO enrichment analysis showing the phosphorylated proteins differentially expressed in microglia from A549-BrM and control group are involved in various cellular pathways.

D. GO enrichment analysis showing the phosphorylated proteins differentially expressed in microglia from A549-BrM and A549 group are involved in various cellular pathways.
