# Supplementary material for: Lung cancer cells upregulate stearoyl-CoA desaturase 1 in microglia by activating the STAT3 pathway to change microglial inflammatory response in lung-to-brain metastases
Source: Cell Death Dis. 2025 Oct 6;16(1):702. doi: 10.1038/s41419-025-08003-2 (PMC12500914; doi:10.1038/s41419-025-08003-2)
Supplement: Supplementary file 5 — western blot original images [file 41419_2025_8003_MOESM5_ESM.docx]

**1. Figure 3 B: SCD1**

**
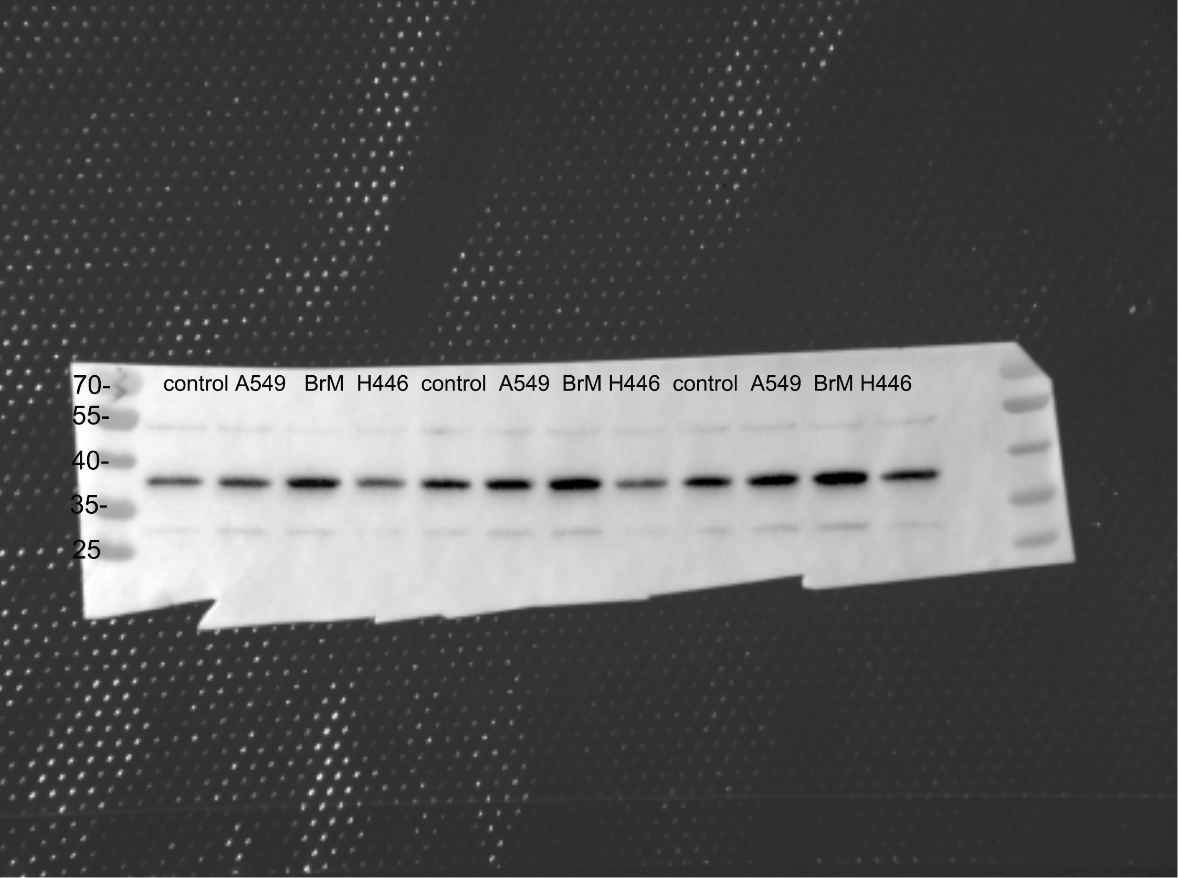
**

**2. Figure 3B: βactin**

**
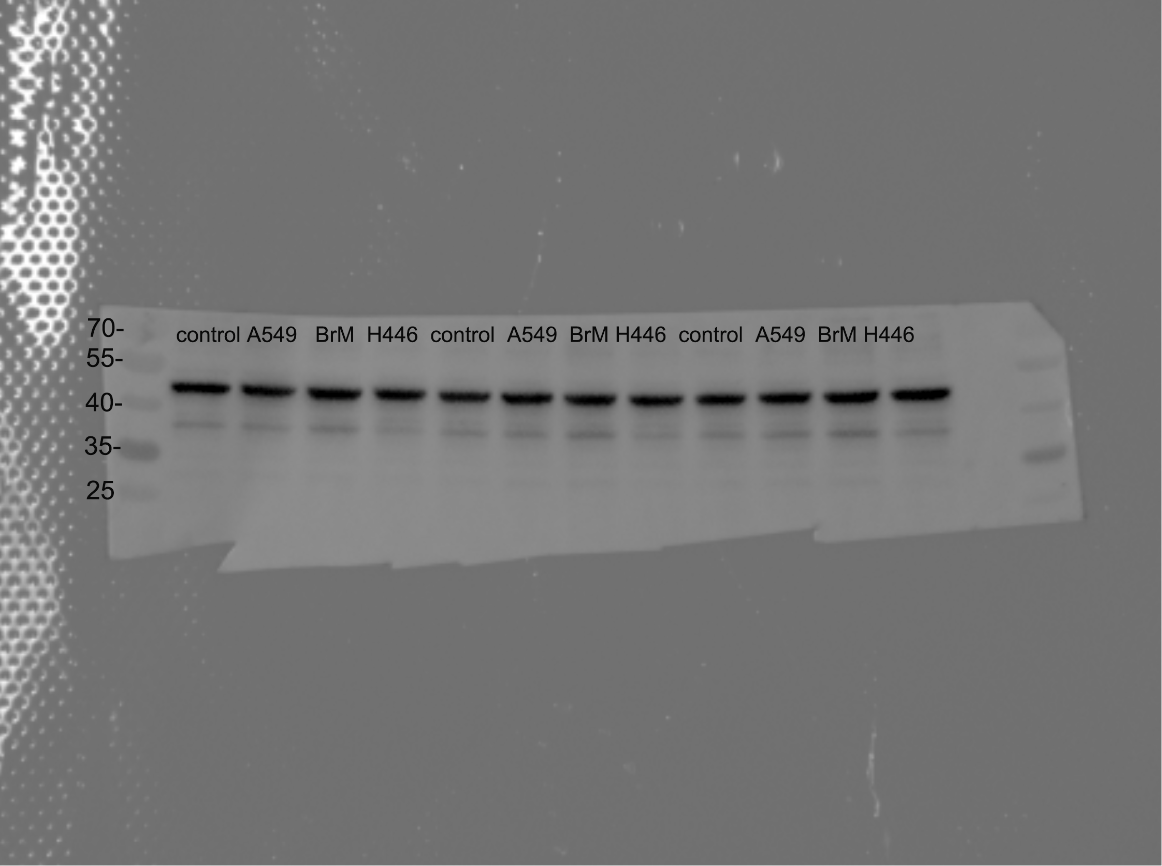
**

**3. Figure 6 C: STAT3**

**
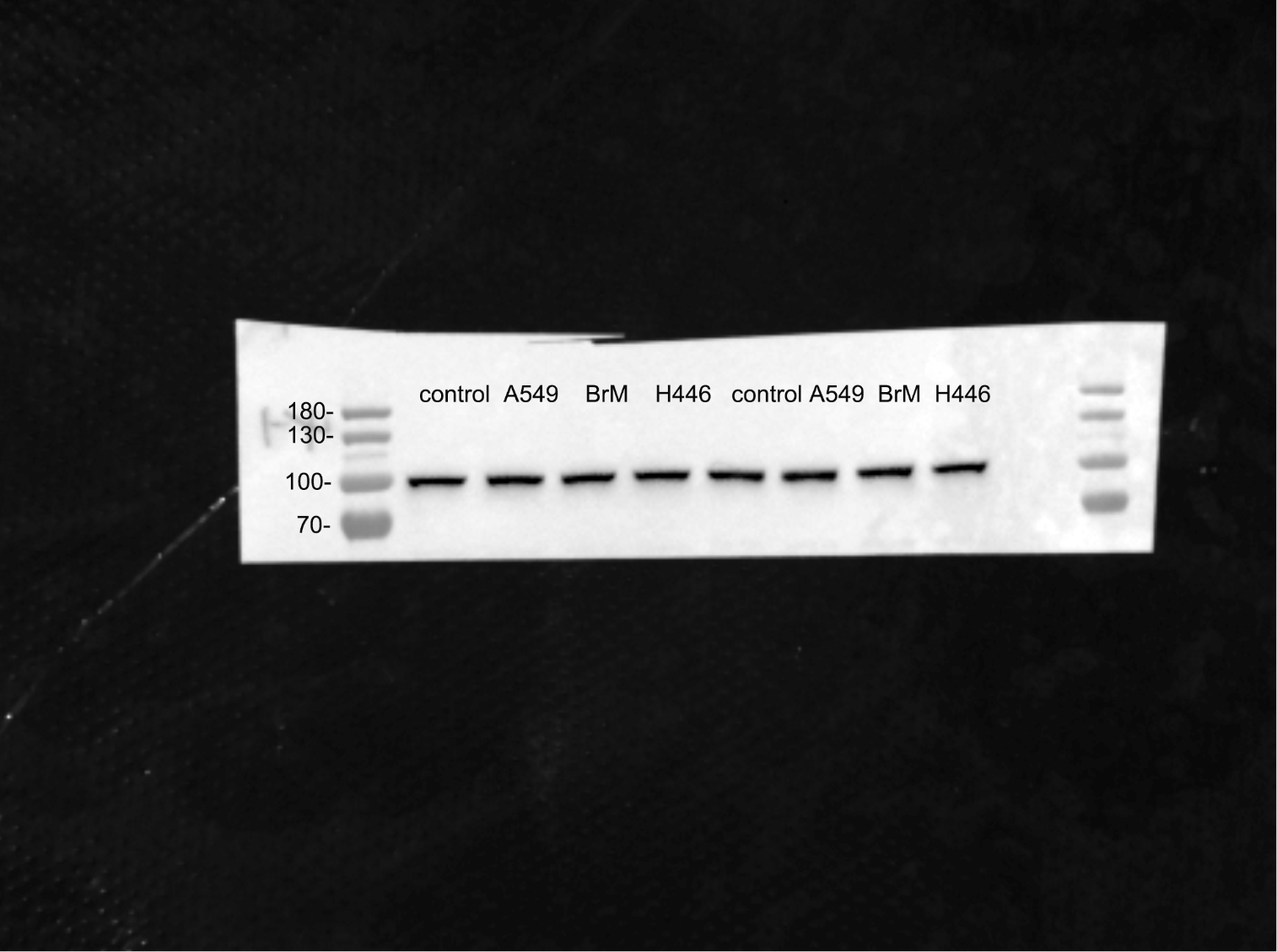
**

**4. Figure 6 C: p-STAT3**

**
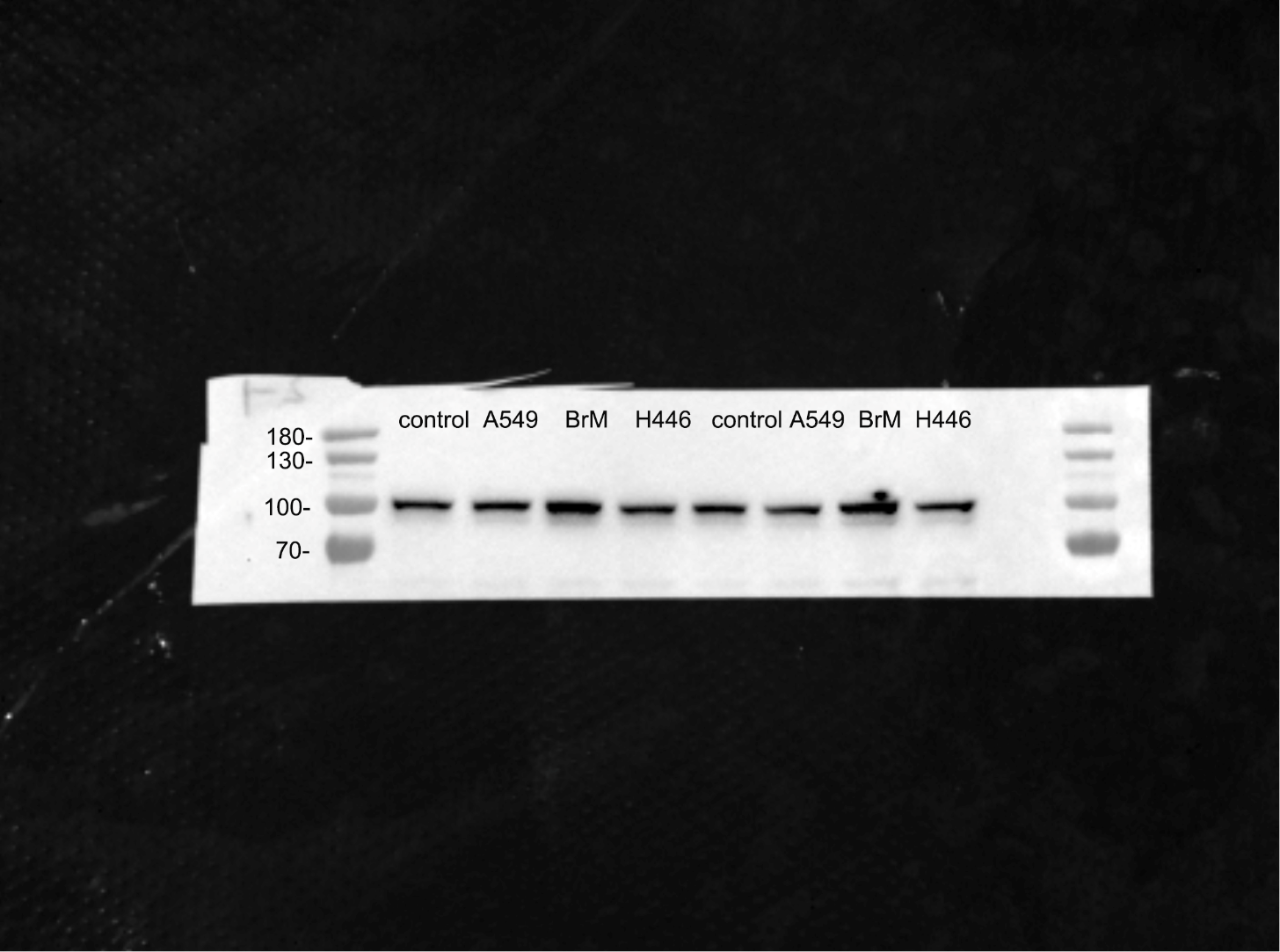
**

**5.Figure 6 C: βactin**

**
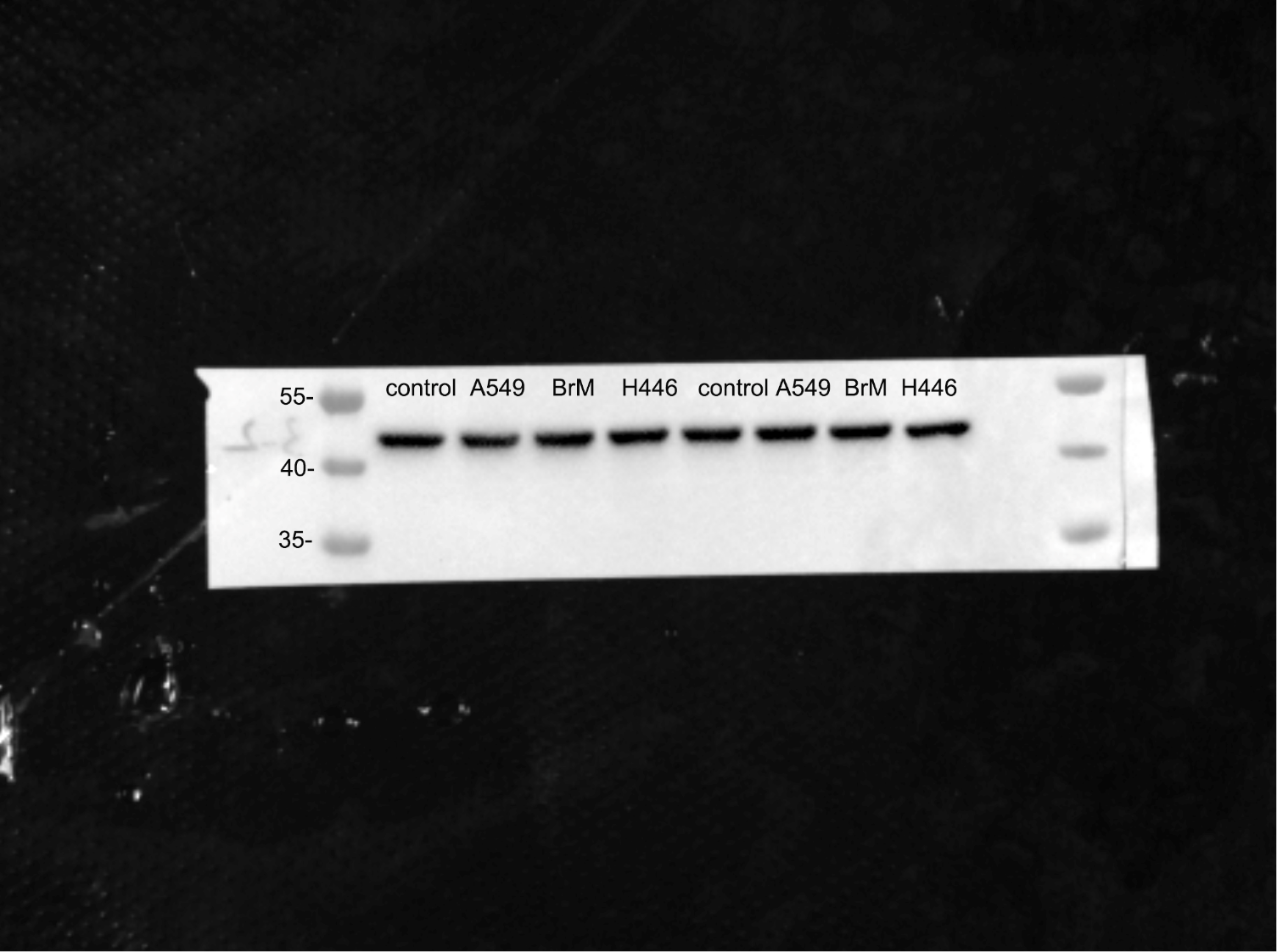
**

**6. Figure 6 D, F: SCD1**

**
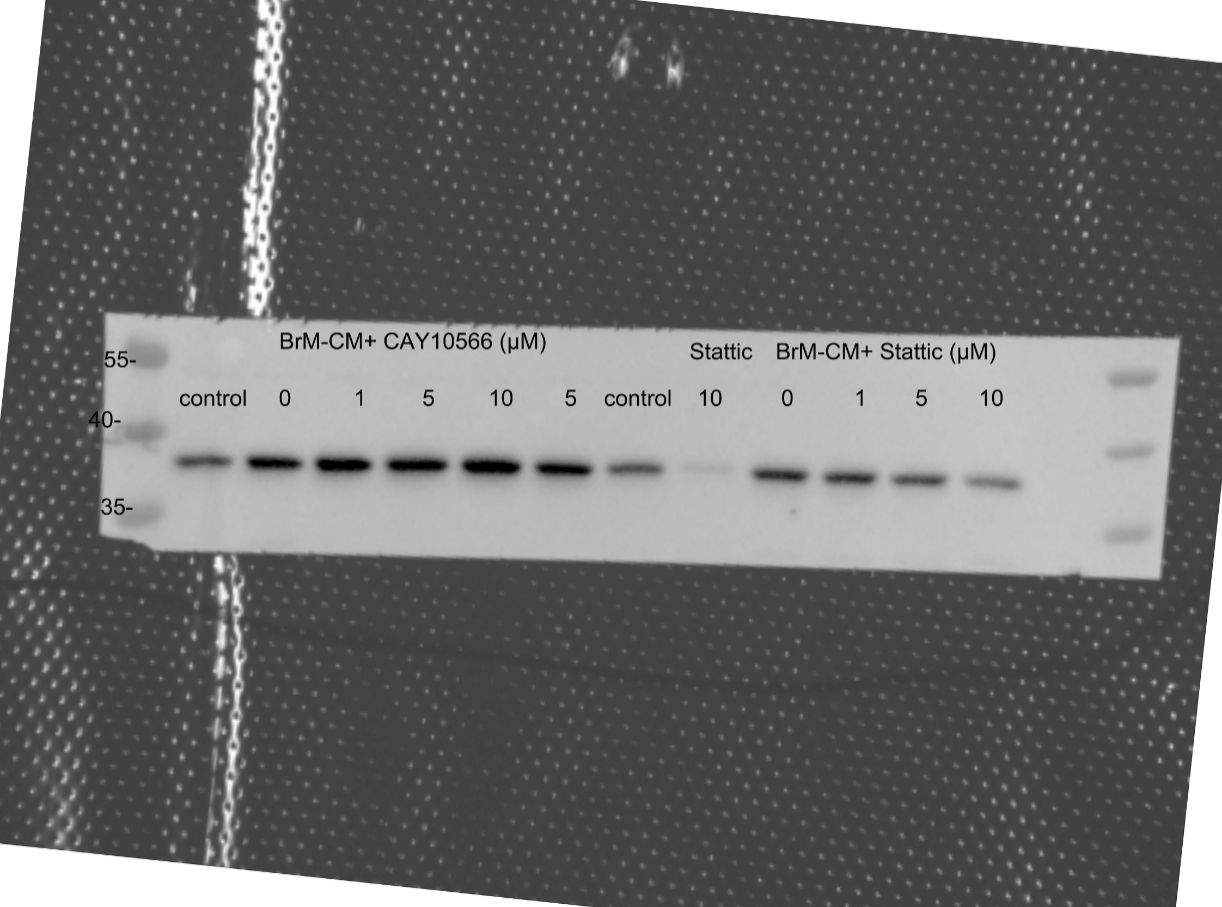
**

**7. Figure 6 D, F: p-STAT3**

**
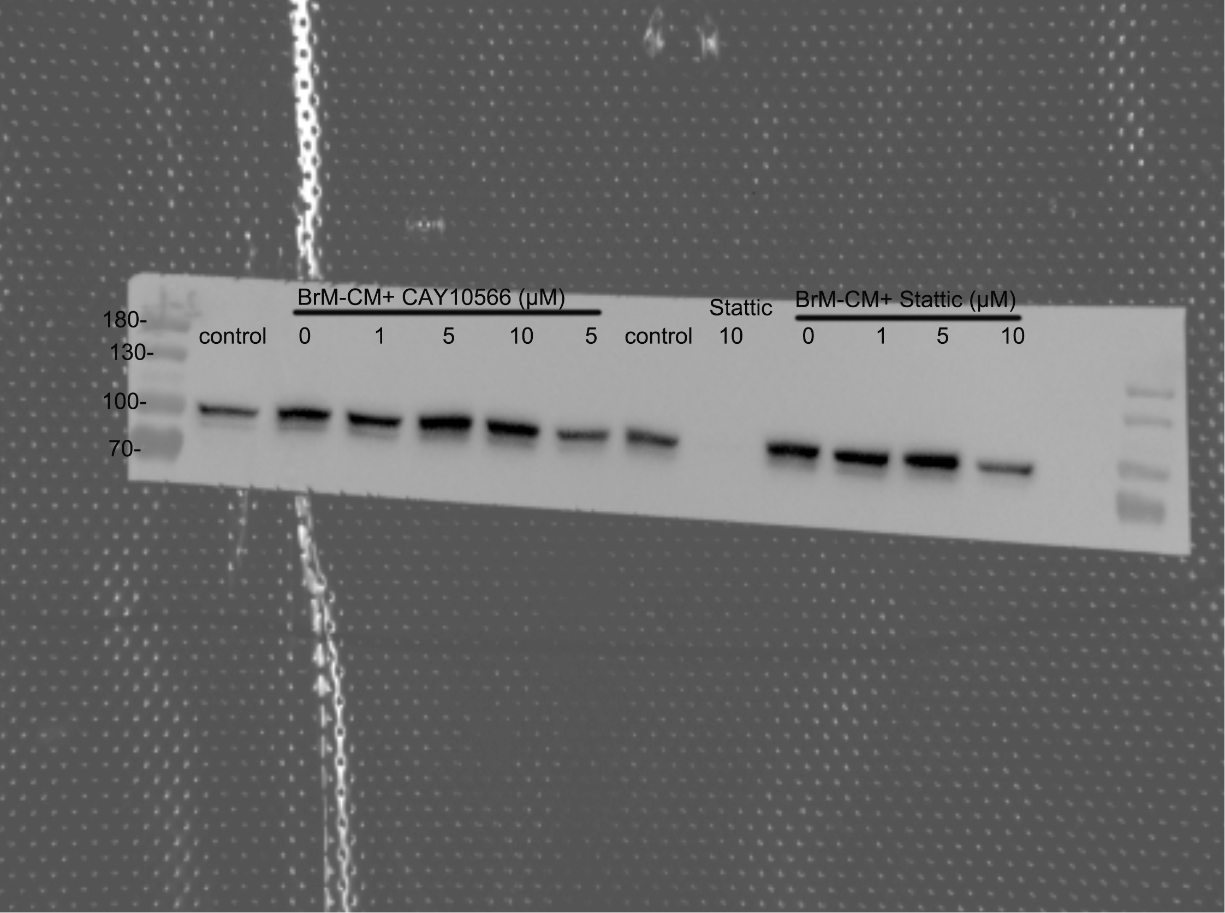
**

**8. Figure 6 D, F: STAT3**

**
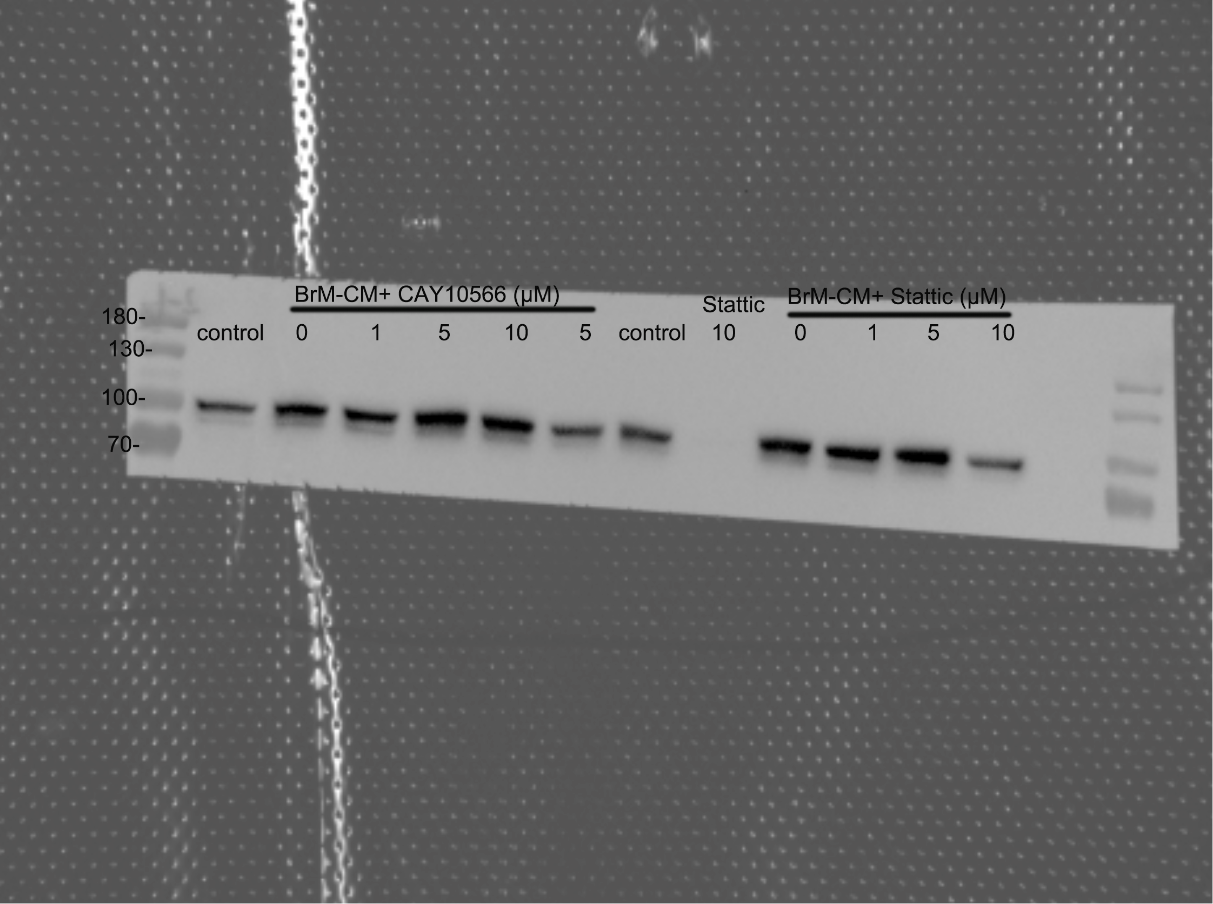
**

**9. Figure 6 D, F: βactin**

**
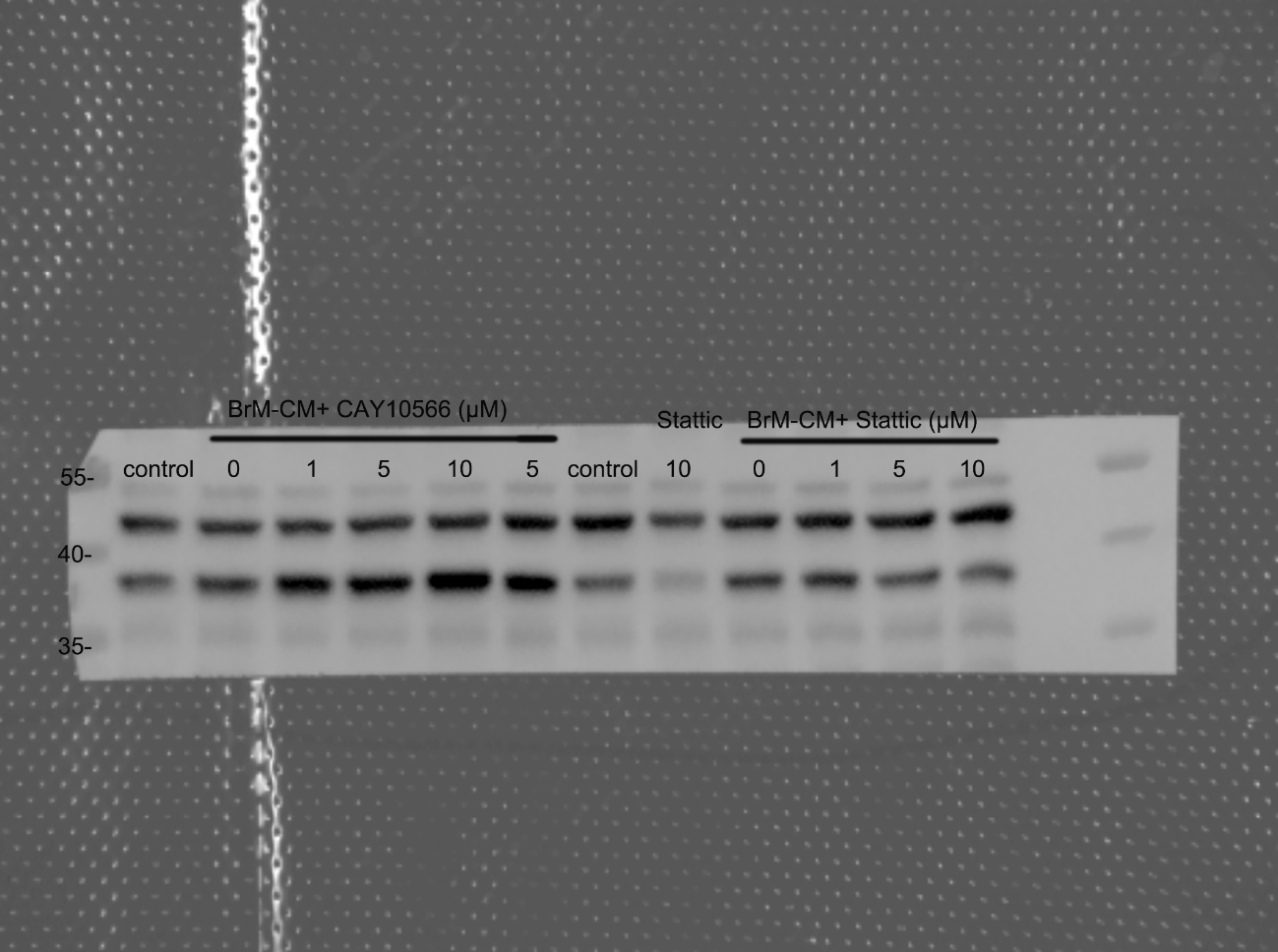
**
